# Interferon-γ Inhibits Ebola Virus Infection

**DOI:** 10.1371/journal.ppat.1005263

**Published:** 2015-11-12

**Authors:** Bethany A. Rhein, Linda S. Powers, Kai Rogers, Manu Anantpadma, Brajesh K. Singh, Yasuteru Sakurai, Thomas Bair, Catherine Miller-Hunt, Patrick Sinn, Robert A. Davey, Martha M. Monick, Wendy Maury

**Affiliations:** 1 Department of Microbiology, The University of Iowa, Iowa City, Iowa, United States of America; 2 Department of Internal Medicine, The University of Iowa, Iowa City, Iowa, United States of America; 3 Department of Virology and Immunology, Texas Biomedical Research Institute, San Antonio, Texas, United States of America; 4 Department of Pediatrics, The University of Iowa, Iowa City, Iowa, United States of America; 5 Iowa Institute for Human Genetics, The University of Iowa, Iowa City, Iowa, United States of America; Thomas Jefferson University, UNITED STATES

## Abstract

Ebola virus outbreaks, such as the 2014 Makona epidemic in West Africa, are episodic and deadly. Filovirus antivirals are currently not clinically available. Our findings suggest interferon gamma, an FDA-approved drug, may serve as a novel and effective prophylactic or treatment option. Using mouse-adapted Ebola virus, we found that murine interferon gamma administered 24 hours before or after infection robustly protects lethally-challenged mice and reduces morbidity and serum viral titers. Furthermore, we demonstrated that interferon gamma profoundly inhibits Ebola virus infection of macrophages, an early cellular target of infection. As early as six hours following *in vitro* infection, Ebola virus RNA levels in interferon gamma-treated macrophages were lower than in infected, untreated cells. Addition of the protein synthesis inhibitor, cycloheximide, to interferon gamma-treated macrophages did not further reduce viral RNA levels, suggesting that interferon gamma blocks life cycle events that require protein synthesis such as virus replication. Microarray studies with interferon gamma-treated human macrophages identified more than 160 interferon-stimulated genes. Ectopic expression of a select group of these genes inhibited Ebola virus infection. These studies provide new potential avenues for antiviral targeting as these genes that have not previously appreciated to inhibit negative strand RNA viruses and specifically Ebola virus infection. As treatment of interferon gamma robustly protects mice from lethal Ebola virus infection, we propose that interferon gamma should be further evaluated for its efficacy as a prophylactic and/or therapeutic strategy against filoviruses. Use of this FDA-approved drug could rapidly be deployed during future outbreaks.

## Author Summary

Filovirus outbreaks occur sporadically, but with increasing frequency. With no current approved filovirus therapeutics, the 2014 Makona Ebola virus epidemic in Guinea, Sierra Leone and Liberia emphasizes the need for effective treatments against this highly pathogenic family of viruses. The use of this FDA-approved drug to inhibit Ebola virus infection would allow rapid implementation of a novel antiviral therapy for future crises. Interferon gamma elicits an antiviral state in antigen-presenting cells and stimulates cellular immune responses. We demonstrate that interferon gamma profoundly inhibits Ebola virus infection of macrophages, which are early cellular targets of Ebola virus. We also identify novel interferon gamma-stimulated genes in human macrophage populations that have not been previously appreciated to inhibit filoviruses or other negative strand RNA viruses. Finally and most importantly, we show that interferon gamma given 24 hours prior to or after virus infection protects mice from lethal Ebola virus challenge, suggesting that this drug may serve as an effective prophylactic and/or therapeutic strategy against this deadly virus.

## Introduction

Ebola virus (EBOV) is a member of the genus *Ebolavirus* within the *Filoviridae* family of highly pathogenic viruses. These viruses cause a severe hemorrhagic fever syndrome in humans and non-human primates (NHP). EBOV infection frequently is associated with high mortality rates and is responsible for the devastating 2014 West African EBOV outbreak [1, 2]. This outbreak has generated a renewed emphasis on the development and approval of safe, effective prophylactics and therapeutics against the virus.

Macrophages and dendritic cells (DCs) play an important role in EBOV pathogenesis as sites of early and sustained virus replication [3, 4]. EBOV infection causes dysregulation of these antigen-presenting cells, increasing production and release of pro-inflammatory proteins, vasoactive molecules, and coagulation factors [5–7]. Pro-inflammatory molecules recruit other target cells to the site of infection, providing additional cells for virus infection and increasing circulation of inflammatory cells and proteins. This uncontrolled amplification of infection and cytokine production results in dysregulation of the inflammatory response, leading to the systemic spread of the virus, excessive cytokine accumulation and circulatory collapse observed in cases of fatal EBOV hemorrhagic fever in humans and non-human primates [3, 4, 6, 8]. Contributing to this amplifying dysregulation, EBOV sustains replication in macrophages and DCs by counteracting early innate immune responses, thereby decreasing effective host responses to the virus [5, 9]. These events in combination with decreased T cell numbers observed in EBOV-infected individuals [10] are thought to lead to poor adaptive immune responses to infection.

Disruption of EBOV infection in macrophages would be predicted to decrease virus loads and associated virus-induced cytokine dysfunction. One approach to controlling virus replication in macrophages is to elicit early innate immune responses. If these responses could be triggered prior to virus-mediated inhibition of these responses, systemic control of EBOV replication should be possible. Previous studies have investigated the ability of type I interferons (IFNs) to decrease EBOV morbidity and mortality with mixed results [11–13]. Jahrling et al. demonstrated that administration of IFN-alpha2b by itself did not significantly alter the course of EBOV in cynomolgus macaques, but more recently IFN-β was shown to prolong survival in rhesus macaques [11, 12]. Additionally, the combination of type I IFN with three monoclonal antibodies (mAbs) against EBOV glycoprotein (GP) provided robust protection against lethal challenge, while neither the IFN nor mAbs alone was highly efficacious [13].

The ability of IFNs other than type I IFN to control EBOV infection has not been explored and several lines of reasoning suggest that type II IFN, interferon gamma (IFNγ), would inhibit EBOV infection of macrophages. Macrophages treated with IFNγ alone or in combination with tumor necrosis factor alpha (TNFα) are activated towards a M1 phenotype that is characteristically proinflammatory and antiviral, enhancing host defenses [14–16]. As IFNγ directly stimulates the expression of a number of interferon-stimulated genes (ISGs) having antiviral activity [17, 18], IFNγ treatment would be predicted to generate macrophages that are resistant to EBOV infection. Since it is thought that EBOV infection of macrophages is responsible for dysregulated cytokine production, reducing infection would likely reduce aberrant cytokine production concomitantly. Finally, as IFNγ potently activates T cell responses through enhancement of phagocytosis and antigen presentation [19], such stimulation of antigen presentation would be predicted to enhance adaptive immune responses to *in vivo* infection.

Here, we demonstrate that IFNγ blocks EBOV infection of murine peritoneal macrophages and robustly protects mice from fatal EBOV infection when administered as late as 24 hours following infection. We also identified novel IFNγ-stimulated genes that inhibit EBOV infection, defining new downstream mechanisms through which this FDA-approved drug functions. During these studies, we evaluated IFNγ inhibition of a BSL2 model virus of EBOV, recombinant vesicular stomatitis virus (VSV) encoding EBOV glycoprotein (EBOV GP/rVSV), in interferon α/β receptor knock out (IFNAR^-/-^) mice. IFNγ was highly effective at inhibiting infection by this recombinant virus. Comparative use of this recombinant BSL2 virus in parallel with EBOV and wild-type VSV permitted us to evaluate the mechanism of IFNγ inhibition in greater detail. These latter findings suggest that IFNγ may have efficacy not only against EBOV, but also a broader range of *Mononegavirales*.

## Results

### IFNγ blocks EBOV and EBOV GP/rVSV infection of primary macrophages

**Fig 1:**
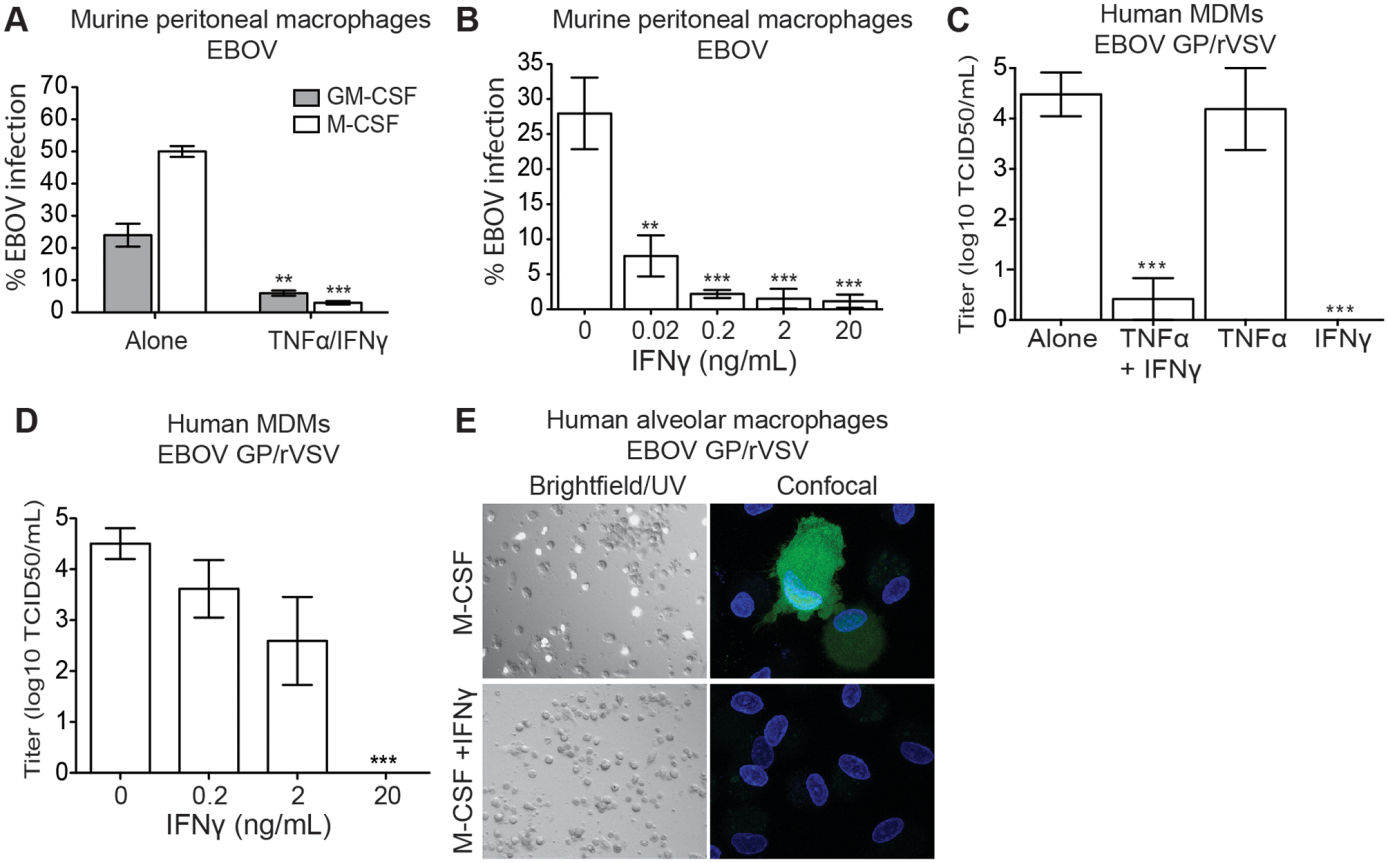
IFNγ-treated macrophages are resistant to EBOV infection. (A) IFNγ/TNFα treatment inhibits EBOV infection of BALB/c peritoneal macrophages. Cultures maintained in media containing GM-CSF or M-CSF were treated with murine IFNγ/TNFα 24 hours prior to EBOV-GFP infection (MOI = 0.05 in Vero E6 cells). Shown are the percent GFP positive cells at 24 hours following infection. (B) IFNγ inhibits EBOV in a dose-dependent manner. BALB/c peritoneal macrophages were infected with EBOV-GFP (MOI = 0.05) 24 hours following IFNγ treatment. (C) IFNγ, but not TNFα, treatment of M-CSF differentiated hMDM cultures inhibits EBOV GP/rVSV infection. Virus titers were determined by end-point dilution on hMDMs from 4 independent donors. (D) Dose response curve of IFNγ inhibition of EBOV GP/rVSV infection of M-CSF-treated hMDMs. (E) IFNγ inhibits EBOV GP/rVSV infection in M-CSF cultured human alveolar macrophages. Left, bright field/UV (magnification: 10X). Right, confocal images (magnification: 100X). All infections were assessed 24 hours following addition of virus. Data are shown as means ± s.e.m. Significance was determined by Student’s t-test compared to no IFNγ control, **p < 0.01, ***p < 0.001.

Mouse peritoneal macrophages treated for 48 hours with either granulocyte-macrophage colony stimulating factor (GM-CSF) or macrophage colony stimulating factor (M-CSF) support robust infection by a recombinant EBOV (formerly Zaire EBOV) that expresses green fluorescent protein (GFP) [20] (Fig 1A). M-CSF-treated cells were consistently more permissive for EBOV infection than GM-CSF-treated cells. Consequently, M-CSF-treated macrophages were primarily investigated in these studies. We observed that M-CSF-treated peritoneal macrophages cultures that were also pretreated with a combination of IFNγ and TNFα were highly resistant to EBOV infection (Fig 1A). The addition of cytokines TNFα and IFNγ to macrophages has been shown to generate a proinflammatory M1 phenotype [14, 15]. Evidence that the combination of these cytokines or IFNγ alone elicited an M1 phenotype of our isolated macrophages included significant increases in expression of proinflammatory genes such as IL-6, TNFα, and CXCL10 in our cells in the presence or absence of EBOV infection (S1 Fig).

Since experiments under BSL4 containment are hazardous, difficult and expensive, the BSL2 model virus, recombinant infectious vesicular stomatitis virus (rVSV) expressing EBOV GP (EBOV GP/rVSV), is frequently used to study a number of aspects of filovirus biology [21–25]. Infection of peritoneal macrophages with EBOV GP/rVSV resulted in remarkably similar effects of GM-CSF, M-CSF and the combination IFNγ/TNFα treatments to that we observed with EBOV (compare Fig 1A–1B and S2 Fig). Thus, throughout these investigations, many of our studies were initially performed with EBOV GP/rVSV and subsequently confirmed and extended with EBOV in a BSL4 setting.

Using EBOV GP/rVSV, we next sought to assess the relative contribution of IFNγ and TNFα in inhibiting virus infection. While TNFα treatment of M-CSF-treated peritoneal macrophages did not inhibit EBOV GP/rVSV replication, the addition of IFNγ prevented infection in either BALB/c or C57BL/6 peritoneal macrophages (S2 Fig). Thus, subsequent studies solely focused on the impact of IFNγ on infection. To confirm that IFNγ, and not TNFα, was important for inhibiting EBOV infection, increasing concentrations of IFNγ were evaluated for virus inhibition. Murine IFNγ reduced EBOV infection in a dose-dependent manner with 20 pg/mL of IFNγ inhibiting more than 70% of infection and 2 ng/mL providing greater than 95% protection (Fig 1B).

The ability of IFNγ, but not TNFα, to inhibit virus infection was also demonstrated in human macrophages. In these studies, six-day M-CSF matured human monocyte derived macrophages (hMDMs) were treated for 24 hours prior to infection with TNFα and/or IFNγ. Similar to our murine macrophages findings, M-CSF-matured hMDMs were highly permissive for EBOV GP/rVSV infection and human IFNγ, but not TNFα, effectively and profoundly blocked the number of cells infected by our recombinant virus (Fig 1C). In a dose-dependent manner, 200 pg/mL reduced EBOV GP/rVSV replication by about 10-fold and 20 ng/mL reduced titers as assessed by end point dilution by more than four orders of magnitude (Fig 1D). In addition, we determined if human IFNγ inhibits EBOV GP/rVSV infection of an *in vivo* matured macrophage population, human alveolar macrophages. These cells were isolated by bronchial lavage and phenotyped as previously described [26, 27]. Similar to the MDM cultures, M-CSF-treated cells were permissive for virus, whereas the addition of IFNγ prevented infection of these cells (Fig 1E).

### IFNγ inhibition requires signaling through the type II interferon receptor in mouse macrophages

IFNγ signals through the type II IFN receptor, thereby activating JAK/STAT pathways and initiating the expression of many ISGs [28]. However, low concentrations of type I IFNs can enhance responses to type II IFN and high concentrations can inhibit [29–31]. To assess if the IFNγ antiviral effect on macrophages was independent of type I IFN receptor signaling, peritoneal macrophages were harvested from interferon α/β receptor knockout mice (IFNAR^-/-^) and cultured for 24 hours with M-CSF or M-CSF plus IFNγ prior to EBOV GP/rVSV infection. IFNγ treatment of BALB/c or C57BL/6 IFNAR^-/-^ macrophages inhibited virus infection (S3A and S3B Fig), indicating that IFNγ does not require the presence of a functional type I IFN receptor for its antiviral effect. To verify that the IFNγ response required the type II receptor, C57BL/6 IFNγ receptor knockout (IFNγR^-/-^) peritoneal macrophages were harvested and stimulated with M-CSF or M-CSF plus IFNγ prior to infection. As expected, IFNγ treatment had no effect on EBOV GP/rVSV viral titers in IFNγR^-/-^ cells, indicating that the type II IFN receptor is required for IFNγ-mediated inhibition of virus infection (S3C Fig).

While studies above indicated that IFNγ blocked EBOV and EBOV GP/rVSV infection of macrophages from wild-type mice, EBOV GP/rVSV infection of these cells was modest and virus spread in these cultures was not observed. In contrast, macrophages from BALB/c or C57BL/6 IFNAR^-/-^ mice were highly permissive to EBOV GP/rVSV and IFNγ treatment of these cells profoundly decreased infection in a dose dependent manner (S3A and S3B Fig). Hence, we used peritoneal macrophages from IFNAR^-/-^ mice in subsequent studies with EBOV GP/rVSV.

### IFNγ inhibition of EBOV RNA levels in infected mouse macrophages

**Fig 2:**
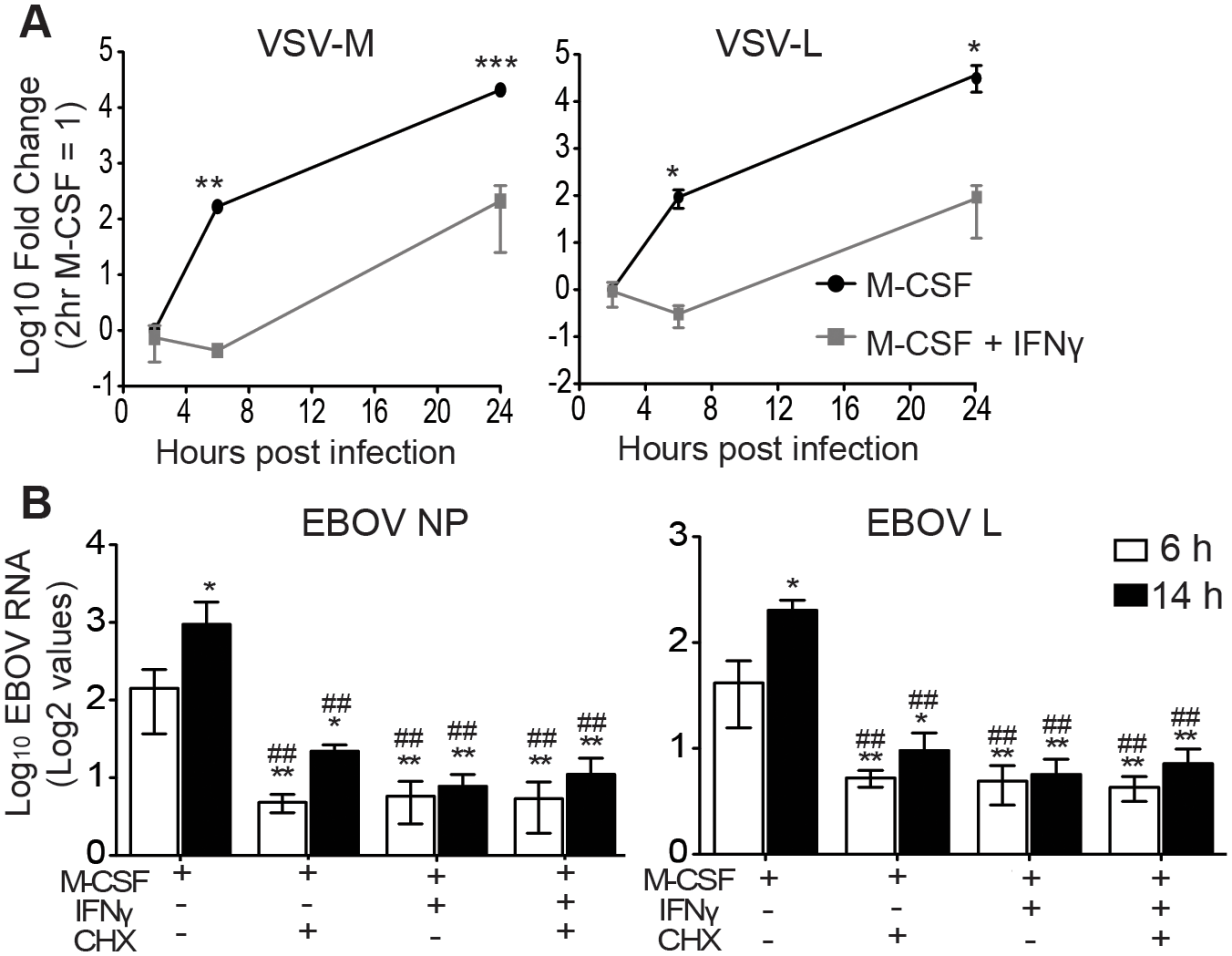
IFNγ blocks EBOV GP/rVSV and EBOV RNA synthesis. (A) M-CSF cultured peritoneal macrophages pre-treated for 24 hours with IFNγ have decreased EBOV GP/rVSV RNA synthesis at 6 and 24 hours following infection. Total RNA from IFNγ-treated or untreated peritoneal macrophages at indicated time points was assessed by qRT-PCR for VSV matrix (M) and polymerase (L) RNA. Log2 value of 2 hour M-CSF alone = 1 and data is shown as Log10 of the fold change. Significance was determined by one sample t-test compared to 2 hour M-CSF only control. *p < 0.05, **p < 0.01, ***p < 0.001. (B) IFNγ treatment decreases EBOV RNA levels to the same extent as the protein synthesis inhibitor cycloheximide. Total RNA isolated at 6 or 14 hours following EBOV infection of M-CSF cultured peritoneal macrophages treated with or without IFNγ and/or CHX was quantified by qRT-PCR for EBOV nucleoprotein (NP) or polymerase (L) RNA. Data is shown as log2 values. Significance was determined by ANOVA with a Tukey post-test. *p < 0.05, **p < 0.01 (compared to 6 hours M-CSF alone). ##*p* < 0.01 (compared to 14 hours M-CSF alone). ns, not significantly different.

To identify the step(s) of the virus life cycle that is/are blocked by IFNγ, BALB/c IFNAR^-/-^ peritoneal macrophages were treated with IFNγ for 24 hours, infected with EBOV GP/rVSV, and monitored for viral RNA accumulation via qRT-PCR. After 2 hours of infection, IFNγ treatment had no significant effect on the quantity of VSV matrix (M) or polymerase (L) RNA detected, suggesting that IFNγ does not affect early events in the life cycle, such as entry (Fig 2A). A previous study supports this observation, as IFNγ treatment of differentiated hMDMs did not significantly reduce entry of EBOV viral-like particles (VLPs) [32]. However, by 6 and 24 hours after infection, the steady increase in VSV M and L RNA levels in infected M-CSF-stimulated peritoneal macrophages was significantly reduced by IFNγ treatment (Fig 2A).

Gene expression of *Mononegavirales* viruses, including filoviruses and rhabdoviruses, is initiated by primary transcription of mRNAs that does not require new viral protein synthesis [33]. However, subsequent viral genome replication requires viral protein production and replication of the genome is blocked by inhibitors of protein synthesis such as cycloheximide (CHX) [34]. Therefore, to narrow down which step(s) within the life cycle is affected by IFNγ, we compared the ability of IFNγ and CHX to inhibit viral RNA production and determined if the combination of these two drugs inhibited viral RNA levels to a greater extent. BALB/c IFNAR^-/-^ peritoneal macrophages were treated with M-CSF in the presence or absence of IFNγ for 24 hours. At the initiation of EBOV infection under BSL4 conditions, CHX was added and viral RNA levels were assessed at 6 and 14 hours of infection. CHX or IFNγ suppressed EBOV nucleoprotein (NP) and polymerase (L) RNA levels equally and the combination of inhibitors did not further reduce EBOV RNA production (Fig 2B). Similar results were observed with EBOV GP/rVSV infections harvested at 6 hours (S4 Fig). In total, these findings indicate that IFNγ treatment interferes with RNA synthesis that is dependent upon protein synthesis and suggests that virus replication is blocked.

### Human macrophage gene arrays identify novel IFNγ-stimulated genes that inhibit EBOV infection

**Fig 3:**
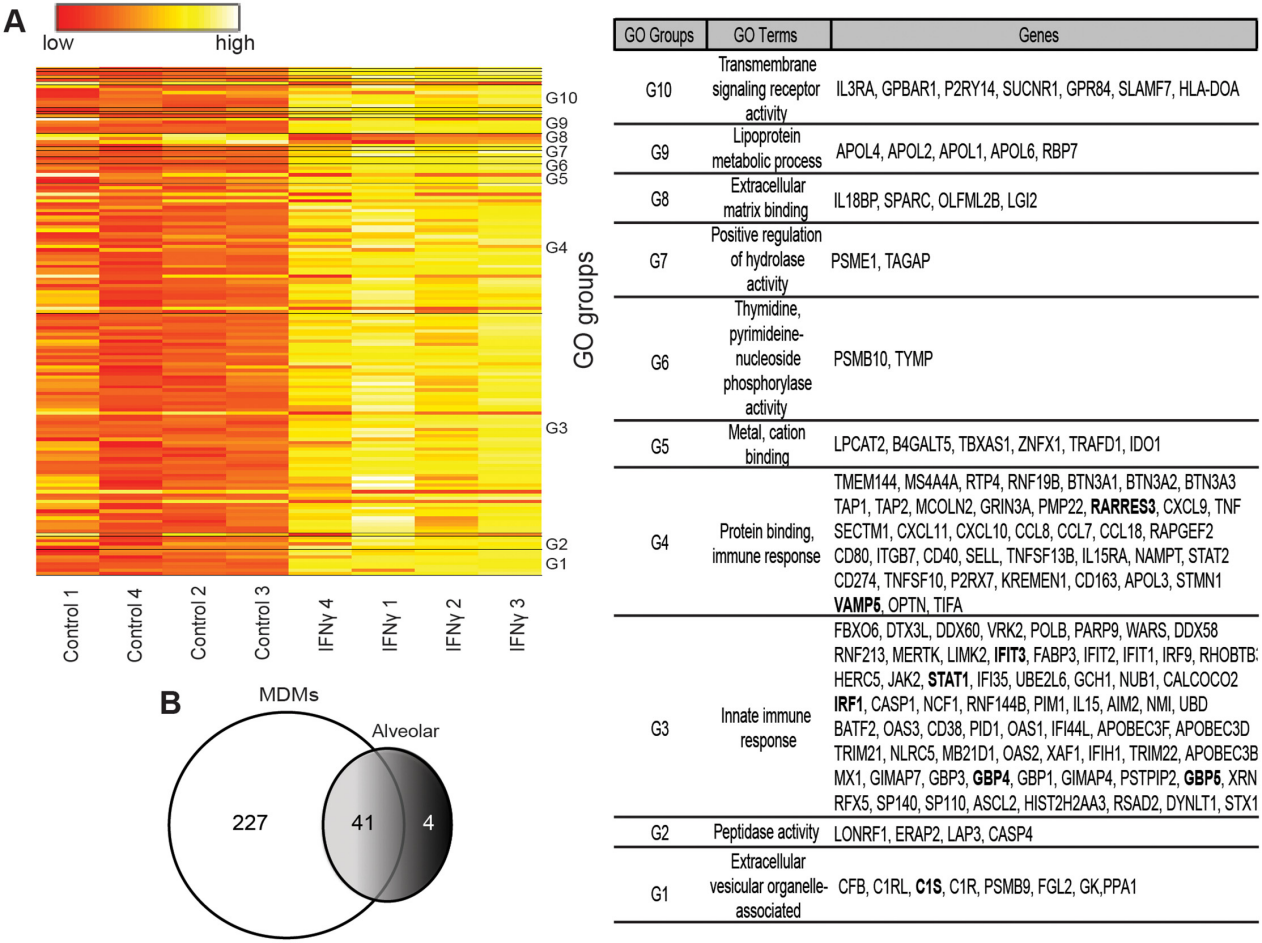
Human MDM gene expression is altered by 24 hours of IFNγ treatment. M-CSF cultured hMDMs were incubated with or without IFNγ for 24 hours and RNA was harvested for gene arrays. (A) Heat map summaries of hMDM gene expression significantly altered by IFNγ. Human MDMs were obtained from four different volunteers and treated with M-CSF or M-CSF plus IFNγ. Genes were clustered into annotated ontology groups and listed in the corresponding GO groups table with their respective GO terms. Significance was determined by paired t-test analysis with cutoff values of at least two-fold change and *p* < 0.01. NUSE analysis of the array demonstrated that the means were centered at a value of 1 and minimum and maximum values between 0.95 and 1.05. ISGs that were assessed further in this study are bolded in the GO table. (B) Venn diagram representing the statistically significant individual and shared genes altered by IFNγ in hMDMs and human alveolar macrophages.

Type I or type II IFN treatment of macrophages elicits expression of hundreds of IFN stimulated genes (ISGs) and suppresses the expression of an additional smaller group of genes. Production of ISG proteins alters macrophage function and protects cells from viral infection [35]. While gene sets activated by these two types of IFNs overlap, both type I and II IFNs also stimulate unique subsets of genes [17, 18, 36]. To determine the impact of IFNγ stimulation on gene expression in our macrophage cultures, we performed microarray analyses. We identified 268 genes with at least two-fold increased or decreased expression in IFNγ-treated six-day-M-CSF-matured hMDM (Fig 3A and S1 Table). In parallel, gene arrays were performed in IFNγ-treated alveolar macrophages and 45 genes were identified to have altered expression (S5 Fig and S2 Table). Forty-one of the significantly altered genes were identified in both arrays (Fig 3B and S6A Fig). Through pathway analysis of the gene profiles, we discovered that the top IFNγ-upregulated genes in our arrays are involved in immune responses, development, signal transduction, ATP metabolism, or transcription (S6B Fig).

**Fig 4:**
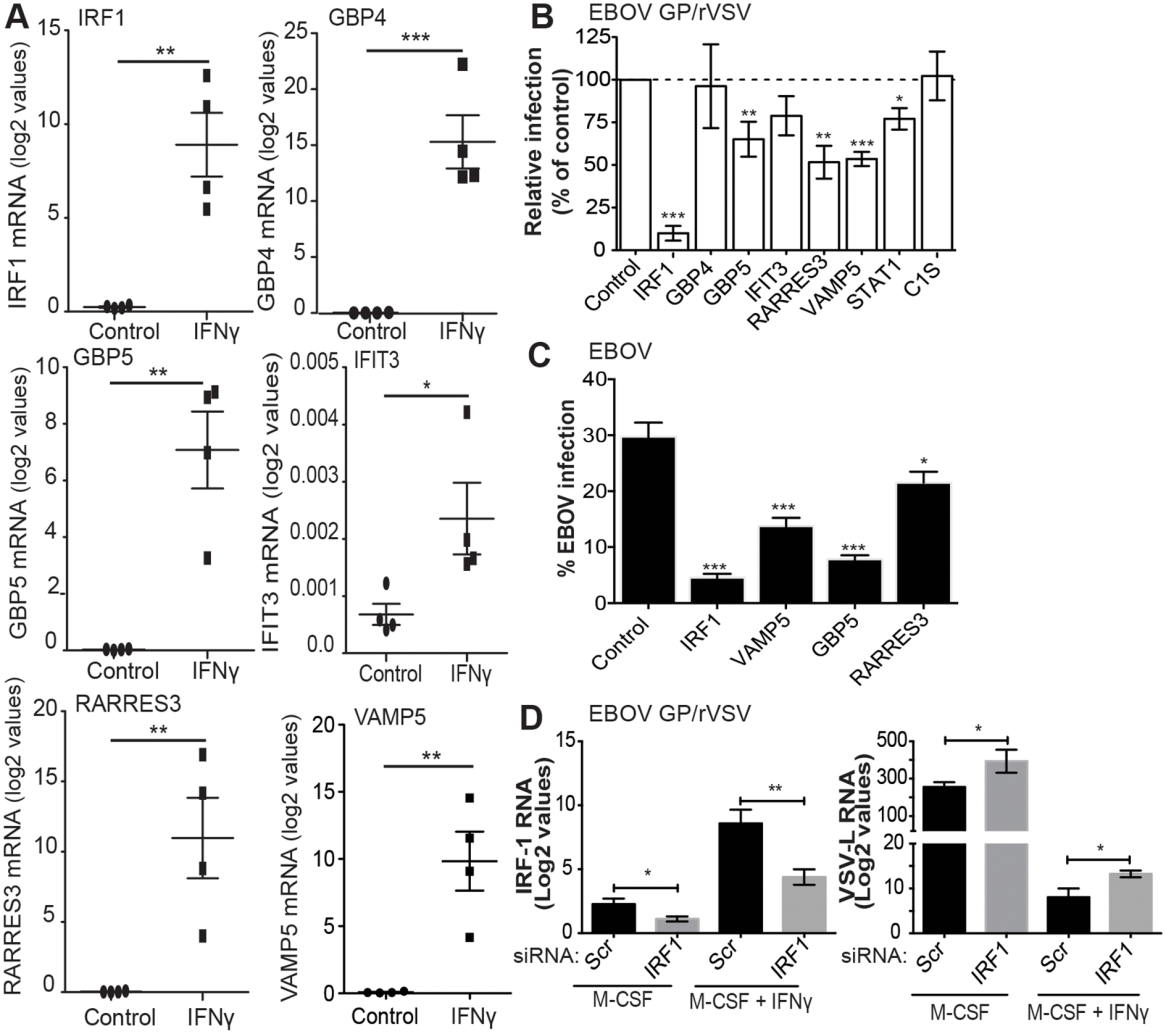
Identification of IFNγ-stimulated genes that inhibit EBOV infection. (A) mRNA validation of hMDM profiling results for several of the top IFNγ-stimulated genes identified in our gene arrays. RNA obtained for the microarray analysis was assessed for mRNA levels of the selected genes by qRT-PCR. Results are represented as the log2 values. (B) Identification of IFNγ-stimulated genes that inhibit EBOV GP/rVSV infection. Highly permissive HEK 293T cells stably expressing TIM -1 (H3 cells) were infected with EBOV GP/rVSV 48 hours following transfection of 2 μg of ISG-RFP lentiviral constructs. Cells gated for RFP expression were assessed for EBOV GP/rVSV infection by detection of GFP. Shown is infection of EBOV GP/rVSV in ISG expressing cells relative to infection of cells transfected with a fluc-RFP expressing lentivirus (control). (C) Novel ISGs that inhibit EBOV GP/rVSV also block EBOV infection. HeLa cells were infected with EBOV 48 hours following electroporation of 5 μg of ISG-RFP lentiviral constructs. Infection was assessed by microscopy 24 hours later and percent of cells that were GFP positive were calculated by CellProfiler image analysis software. A lentiviral construct expressing IRF1 served as a positive control in these studies. (D) IRF1 knock down increases EBOV GP/rVSV infection following IFNγ stimulation. IRF1 or scrambled (Scr) siRNA were loaded into HEK 293T derived exosomes. SiRNA loaded exosomes (2.5 μg) were delivered and IFNγ added to BALB/c IFNAR^-/-^ peritoneal macrophages 24 hours prior to EBOV GP/rVSV infection (MOI = 0.1). Twenty-four hours following infection, total RNA was isolated from the macrophages. Amount of IRF1 expression and infection (by detection of VSV polymerase (L)) was quantified by qRT-PCR. Results represent the means ± s.e.m. Significance was determined by Student’s t-test analysis, *p < 0.05, **p < 0.01, ***p < 0.001.

Our arrays identified IFNγ-enhanced expression of ISGs known to be primary-response genes, such as the transcription factors IRF1 and IRF9, and more poorly studied secondary-response ISGs, such as the p65 GBPs and the apolipoprotein L family of proteins that are thought be regulated by IRFs [37]. Consistent with previous reports, a number of chemokines (CXCL9, CXCL10, CCL8 and CXCL11) and complement components (C1s and C1r) were also highly upregulated by IFNγ [14, 16, 17, 38]. Our array findings were validated by examining mRNA levels of several of the top ISGs, including IRF1, GBP4, GBP5, IFIT3, RARRES3, and VAMP5 by qRT-PCR (Fig 4A and S5B Fig).

To assess the importance of some of our most significantly upregulated ISGs in controlling EBOV GP/rVSV and EBOV infection, IFNγ-upregulated genes were transfected into cells and their effect on virus infection was assessed. The panel of ISGs included both well-studied and poorly characterized IFNγ-responsive genes. Eight lentiviral constructs that expressed an ISG and red fluorescent protein (RFP) were assessed for inhibition of EBOV GP/rVSV or EBOV in highly permissive cells [23].

Transfection of the construct expressing IRF1 strongly inhibited EBOV GP/rVSV and EBOV replication, as has been previously shown for positive strand and other negative strand RNA viruses (Fig 4B and 4C) [39, 40]. As IRF1 is a transcription factor critical for IFNγ signaling by driving downstream expression of many IFNγ target genes [19], we also assessed if knock down of IRF1 in M-CSF-treated and M-CSF/IFNγ-treated macrophages altered infection of EBOV GP/rVSV in peritoneal macrophages. IRF1 siRNA or scrambled control siRNA was delivered to peritoneal macrophages by exosomes since direct siRNA transfection of macrophages is highly inefficient. To validate this siRNA delivery method, we initially demonstrated that exosomes are efficiently engulfed by murine peritoneal macrophages (S7 Fig). In the context of EBOV GP/rVSV infection, delivery of IRF1 siRNA containing exosomes knocked down IRF1 mRNA levels by approximately 50% in both M-CSF- and M-CSF/IFNγ-treated macrophages (Fig 4D). With IRF1 knock down, a significant increase in EBOV GP/rVSV RNA was observed 24 hours following infection, consistent with the importance of IRF1 expression in controlling virus infection by IFNγ.

Additional less well-characterized ISGs that were highly upregulated in our gene array studies were also assessed for their ability to directly control virus infection. Ectopic expression of GBP5, RARRES3 and VAMP5 resulted in significant inhibition of EBOV GP/rVSV (Fig 4B). Evaluation of these ISGs for their ability to inhibit EBOV demonstrated that they also effectively blocked EBOV, identifying these as novel ISGs that are likely important for the control of EBOV in IFNγ-treated cells. Surprisingly, expression of STAT1 only modestly inhibited EBOV GP/rVSV infection, likely because significant levels of STAT1 are constitutively expressed within these cells and control of STAT1 activity is primarily regulated by its phosphorylation status [41–43]. Certainly not all ISGs tested that were highly upregulated in our gene arrays inhibited virus replication; GBP4, IFIT3, IFI27 and C1S had no effect on EBOV GP/rVSV.

### *In vivo* IFNγ treatment protects IFNAR^-/-^ mice against lethal EBOV GP/rVSV challenge, but is less efficacious against wild-type VSV

**Fig 5:**
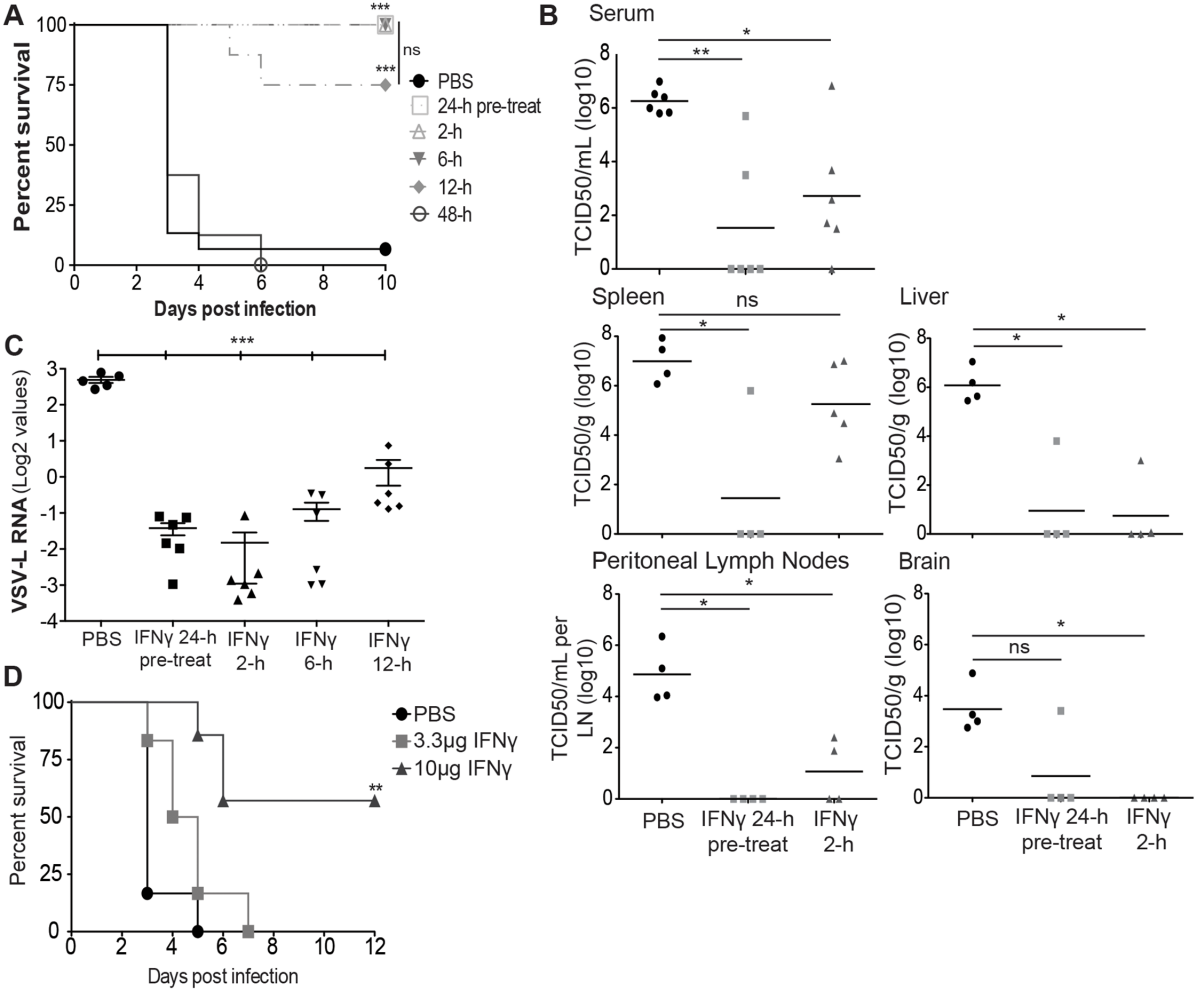
IFNγ reduces EBOV-GP/rVSV morbidity and mortality. (A) IFNγ enhances survival of EBOV GP/rVSV infected mice. IFNγ (10 μg) or PBS was administered by i.p. injection to BALB/c IFNAR^-/-^ mice 24 hours prior to or 2, 6, 12 or 48 hours following EBOV GP/rVSV infection (n≥8/treatment). (B) IFNγ treatment as a 24 hour pre-treatment or a 2 hour post-treatment reduces serum viremia and organ titers of EBOV GP/rVSV-infected mice. Sera and organs were harvested at 48 hours following infection (n≥ 4/treatment) in mice treated with 3.3μg of IFNγ. Viremia and organ virus titers were determined by endpoint dilution of serum or homogenized organ samples on Vero cells. Significance was calculated by Mann-Whitney test compared to PBS control, *p < 0.05, **p < 0.01. ns, not significant. (C) Intraperitoneal IFNγ treatment of mice significantly inhibits EBOV GP/rVSV infection of peritoneal cells. Peritoneal cells were isolated from EBOV GP/rVSV infected mice treated with 3.3μg of IFNγ at times noted prior to or following challenge. Amount of VSV-L RNA was determined by qRT-PCR. Significance was determined by ANOVA with a Tukey post-test, ***p < 0.001. (D) Intramuscular administration of IFNγ increases survival of IFNAR^-/-^ mice. PBS or IFNγ at the indicated concentration was administered by i.m. injection 24 hours prior to i.p. injection of EBOV GP/rVSV. For A & D, significance was determined by Mantel-Cox Test, **p < 0.01, ***p < 0.001.

Since IFNγ robustly inhibits EBOV GP/rVSV and EBOV infection of macrophages and macrophages are an important cellular target for filoviruses pathogenesis, we evaluated the ability of intraperitoneally (i.p.) administered IFNγ to protect mice against lethal i.p. challenge. For these studies, BALB/c IFNAR^-/-^ mice were used as little to no pathogenesis is observed with EBOV GP/rVSV infections of wild-type mice, but significant morbidity and mortality occurs in IFNAR^-/-^ mice. Initial IFNγ dose response studies demonstrated that both 3.3 and 10 μg of IFNγ protected IFNAR^-/-^ mice against lethal EBOV GP/rVSV challenge (S8A Fig). Further, neither dose of IFNγ had any detectable consequence on the weight or health of the mice. Therefore, subsequent studies were performed with both dosages. IFNγ administered by i.p. injection 24 hours prior to, 2 or 6 hours after a lethal dose of EBOV GP/rVSV protected mice against challenge, with treated mice demonstrating significantly reduced mortality, weight loss and clinical sickness scores compared to untreated, virus-infected animals (Figs 5A and S8B–S8D). At the 10 μg IFNγ dose, we also assessed survival of mice when IFNγ was given at 12 and 48 hours after challenge. While only 75% of the mice survived challenge in the 12-hour post challenge group, the protection offered by this treatment was not statistically different than that conferred at time points more proximal to the challenge (Fig 5A). However, IFNγ administered 48 hours following EBOV GP/rVSV did not protect mice against lethal challenge.

Consistent with reduced morbidity and mortality in the EBOV GP/rVSV infected, IFNγ-treated mice, mice treated with 3.3 μg of IFNγ at 24 hour before infection had dramatically lower EBOV GP/rVSV viremia at day 2 of infection (Fig 5B). Little to no detectable viral load was observed in liver, peritoneal lymph nodes or brain, of the pre-treated mice with somewhat higher levels of virus present in the spleen. Similar trends were observed in mice treated with IFNγ 2 hours following EBOV GP/rVSV infection (Fig 5B), but splenic virus loads were higher in these mice, suggesting that some virus had trafficked to the spleen by 2 hours following inoculation. Nonetheless, IFNγ treatment still controlled virus titers in other organs.

To assess the impact of IFNγ treatment on cells at the site of infection, peritoneal cells were isolated 24 hours following EBOV GP/rVSV infection of untreated or IFNγ-treated mice. IFNγ treated peritoneal cells had reduced viral RNA levels compared to infected, PBS-treated control cells, regardless of whether IFNγ was given 24 hours prior to, 2, 6 or 12 hours following challenge. These findings suggest that active infection at the site of challenge is ongoing at 24 hours following infection and that IFNγ administration either before or at early time points following infection is able to profoundly reduce virus load at the site of virus challenge (Fig 5C).

As a control for these studies, we also evaluated the ability of IFNγ to protect against wild-type VSV infection. Initial studies demonstrated that equivalent concentrations of VSV were more virulent in mice than our recombinant virus, EBOV GP/rVSV, perhaps because of the broad cellular tropism conferred by the native VSV glycoprotein G [44, 45]. As a consequence, in these studies we used 10^2^ infectious units of VSV as our challenge virus, the lowest concentration of VSV that resulted in predictable death. IFNAR^-/-^ mice challenged with VSV were given 3.3 μg of IFNγ either 24 hours prior or 2 hours after challenge. While the 24-hour pre-treated mice had significant protection, 40% mortality was still observed. Surprisingly, the survival of the 2-hour IFNγ post-treatment mice was not significantly different from the PBS-treated, VSV infected mice (S8B Fig). This finding stands in contrast to the robust protection IFNγ conferred at 2, 6 and 12 hours following EBOV GP/rVSV challenge. These findings suggested that VSV infection is less sensitive to IFNγ, particularly when administered as a post-challenge antiviral.

Previous studies have demonstrated that IFNγ treatment administered by intramuscular (i.m.) injection allows for slower release and an extended half-life of the drug [46]. Further, this injection route is utilized in many clinical therapeutics. Thus, we investigated the ability of this administration route to protect mice against lethal EBOV GP/rVSV. Mice given 10 μg of IFNγ by i.m. injection 24 hours prior to EBOV GP/rVSV infection significantly protected against lethal challenge (Fig 5D).

### IFNγ protects mice from lethal EBOV challenge

**Fig 6:**
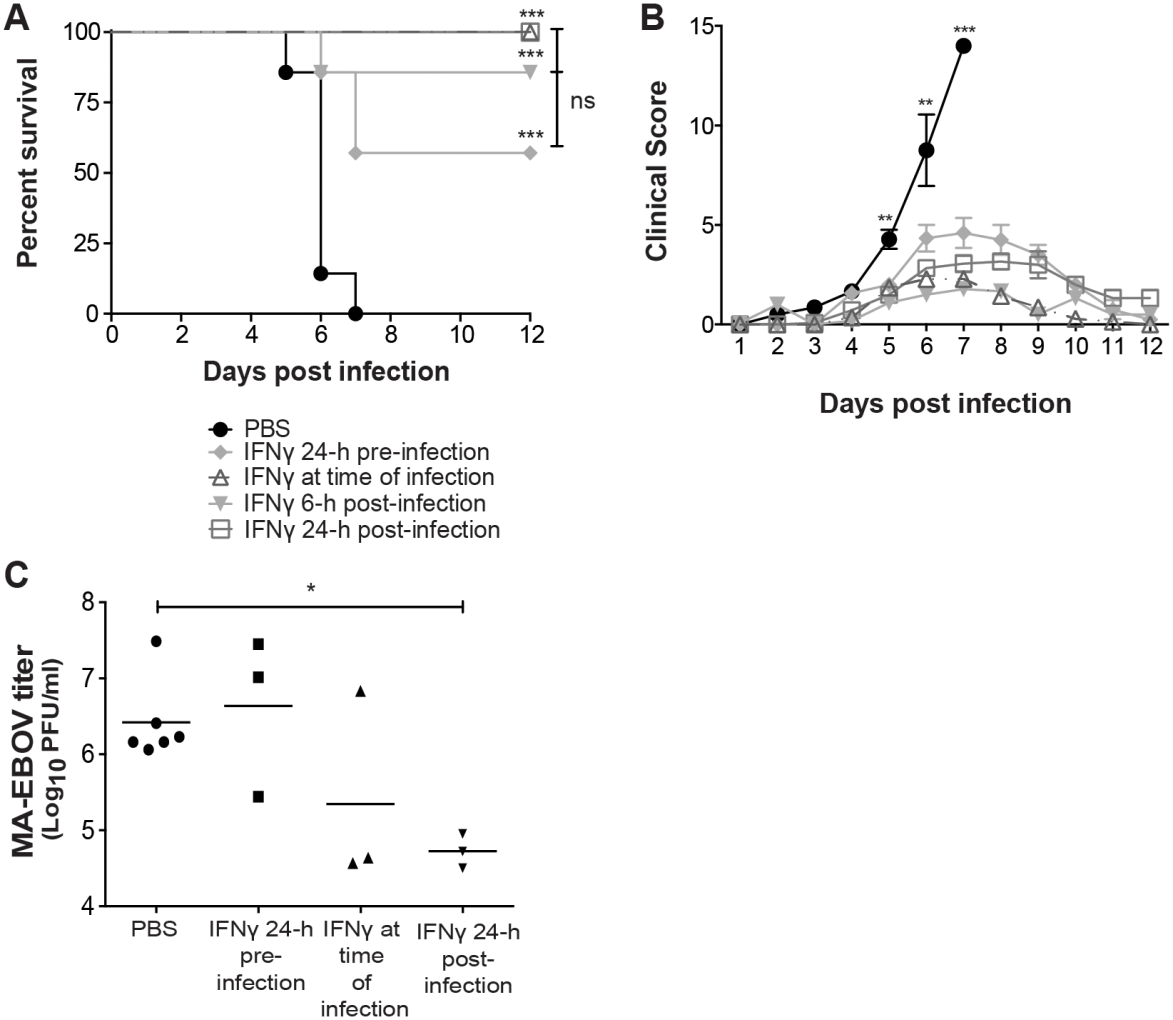
IFNγ protects mice from EBOV morbidity and mortality. (A) IFNγ protects mice from lethal MA-EBOV infection. IFNγ (10 μg) or PBS was administered by i.p. injection to BALB/c mice 24 hours prior to, at the time of infection, 6 or 24 hours following infection (n ≥ 7/treatment). Significance was determined by Mantel-Cox Test, **p < 0.01, ***p < 0.001. ns, not significantly different. (B) IFNγ treatment reduces MA-EBOV morbidity. Results represent mean clinical sickness scores ± s.e.m (n ≥ 7/treatment). Significance was determined by Student’s t-test compared to PBS control. (C) IFNγ treatment significantly reduces MA-EBOV viremia in mice treated 24 hours following infection. Serum was collected 4 days following infection (n = 3–6 mice/treatment) and serial dilution of sera on Vero-E6 cells to assess plaques formed determined viremia titers. Results represent means ± s.e.m. Significance was determined by Mann-Whitney test compared to PBS control, * p < 0.05.

Based on the promising results we observed with IFNγ protection of EBOV GP/rVSV infected mice, we next sought to determine the ability of IFNγ to protect mice from lethal challenge with mouse-adapted EBOV (MA-EBOV). BALB/c mice were administered 3 or 10 μg of IFNγ i.p. and challenged i.p. with MA-EBOV 24 hours later. Initial IFNγ dose response studies indicated that mice pretreated with 10 μg of IFNγ were better protected against MA-EBOV than treatment with 3 μg (S9A Fig). Thus, in subsequent studies, mice challenged with MA-EBOV were administered 10 μg of IFNγ. Since IFNγ given 48 hours following infection did not protect IFNAR^-/-^ mice against EBOV GP/rVSV (Fig 5A), in these studies we assessed the protection IFNγ conferred against MA-EBOV when administered 24 hours prior to, at the time of infection, 6 or 24 hours following infection. All IFNγ-treated mice, regardless of time of treatment, had significantly less morbidity and mortality than untreated, infected mice, with treatment as late as 24 hours following infection protecting 100% of the mice from death (Fig 6A and 6B). Lower MA-EBOV viremia levels were observed in the 24 hour post challenge treated mice, but not in the 24 hour pre-infection treatment group, suggesting that IFNγ administration as a post-exposure antiviral may prove more efficacious than when used prophylactically (Fig 6C).

## Discussion

Our results are the first to demonstrate the ability of IFNγ to protect animals both prophylactically and therapeutically against EBOV infection and suggest that this FDA-approved drug may be a useful antiviral for individuals with recent high-risk exposure. IFNγ treatment profoundly inhibited EBOV infection of peritoneal macrophages in tissue culture, consistent with the protection conferred by IFNγ and evidence that this cell type is an important early target for virus replication. Since antiviral efficacy required the presence of IFNγ receptor, but not the type I receptor, IFNγ control of EBOV infection occurs independently of type I IFN responses. Thus, we sought to identify specific IFNγ-stimulated genes involved in its antiviral effect. In addition to previously characterized ISGs, we identified three novel IFNγ-stimulated factors, GBP5, RARRES3 and VAMP5. To date, GBP5, RARRES3 or VAMP5 has not been shown to control negative strand RNA virus infection. Finally, we demonstrated that the recombinant BSL2-level virus, EBOV GP/rVSV, recapitulates our findings with EBOV, arguing that studies with this BSL2 model virus may serve as a safer and cost effective alternative for initial evaluations of novel anti-filoviral agents.

Antiviral activity conferred by IFNγ against members of the *Mononegavirales* has been previously reported. For instance, addition of *Pteropus alecto* IFNγ to bat cells protects against the paramyxoviruses, Semiki forest virus and Hendra virus [47]. Additionally, several lines of evidence indicate that IFNγ is effective against rabies virus in tissue culture and *in vivo*, potentially through stimulation of type I IFN pathways [48, 49].

Our studies also suggest that IFNγ treatment does not specifically target EBOV, as it protected against our BSL2 recombinant virus EBOV GP/rVSV and, to a more modest degree, against VSV in mice lacking type I IFN signaling. However, the timing of IFNγ protection against wild-type EBOV and wild-type VSV differed. IFNγ treatment protected against VSV only when given prior to infection, whereas post-challenge treatment appeared somewhat more efficacious against EBOV than pre-challenge treatment. Recent studies demonstrate that some ISGs are solely synthesized during the first few hours following IFN stimulation, whereas others are expressed for longer periods of time [50]. The difference in timing of IFNγ protection against VSV versus EBOV suggests that ISGs responsible for protection may differ. Future studies are needed to explore this possibility. Since mice challenged with EBOV GP/rVSV were protected by IFNγ treatment at post challenge time points in a manner similar to EBOV, the difference in timing of protection may be related to the cell populations targeted by the two wild-type viruses.

Our studies have only begun to identify IFNγ-stimulated proteins that contribute to the control of EBOV infection. Studies with type I IFNs have shown that a subset of ISGs preferentially target negative strand RNA viruses [51] and this may be the case with IFNγ-stimulated ISGs as well. Not surprisingly, expression of IFNγ-activated transcription factor IRF1 inhibited EBOV infection and this ISG served as a positive control in these experiments [18, 40, 52]. Other downstream ISGs were selected for study based on the enhancement of their expression elicited by IFNγ in our gene array studies and availability of expression constructs. The ability of GBP5, RARRES3 and VAMP5 to inhibit negative strand RNA viruses has been poorly studied and the mechanisms driving inhibition of virus replication remain to be elucidated. Each of these ISGs reduced EBOV infection; however, as has been shown with other viruses, combinations of ISGs are likely to provide additive or even synergistic inhibition if the ISGs are targeting independent modulatory pathways [40].

We also demonstrated that knockdown of IRF1 could modestly rescue EBOV GP/rVSV infection in peritoneal macrophages that were stimulated with IFNγ. GBP5, RARRES3 and VAMP5 all contain predicted IRF1 binding sites based on analysis of their genomic sequence with the UCSF genome browser. In addition, a recent study demonstrated that murine IRF1^-/-^ bone-marrow derived macrophages have significantly decreased GBP5 gene expression compared to WT cells following *Francisella novicida* infection [53]. Together, these observations suggest IRF1 transcriptional stimulation may control expression of these novel ISGs that in turn participate in control of EBOV infection.

Our identification of IFNγ-elicited ISGs that inhibit EBOV infection adds to the ISGs that are identified to target this virus. To date, a limited number of ISGs has been identified to restrict EBOV infection, including tetherin, ISG15, RIG-I, STAT1 and STAT2 [54–59]. Additionally, IFITM1 has been identified to inhibit EBOV entry. This ISG blocks EBOV GP-dependent entry [60]. Interestingly, in our studies, IFITM1 expression was not significantly upregulated in IFNγ-stimulated macrophages. Consistent with this, we did not see an IFNγ-dependent impact on total viral RNA present in cells at early times during infection (2 hours), suggesting very early events are not affected by IFNγ treatment. Consistent with this, a previous study demonstrated that IFNγ does not affect EBOV GP-dependent entry [32].

IFNγ reduced viral RNA levels in a manner similar to that observed with the protein synthesis inhibitor, cycloheximide, suggesting that IFNγ inhibits one or more viral life cycle events at or downstream of translation. ISGs have previously been identified to block viral protein translation, including the oligoadenylate synthetases [61] and indoleamine 2,3-dioxygenase [62]; however, to date, none of these has been assessed for their ability to inhibit EBOV replication. Expression of both of these ISGs was significantly increased in our IFNγ-stimulated macrophages and likely contributed to the inhibition of EBOV infection that we observed.

IFNγ is produced by several different hematopoietic cell types including natural killer cells, natural killer T cells and T lymphocytes [63, 64]. IFNγ induces specific cytotoxic and antiviral immunity by direct ISG production and through indirect mechanisms that likely provide additional mechanisms of action benefiting its clinical profile [19]. These added effects include IFNγ assisting in the activation of the adaptive immune system. IFNγ/receptor interactions lead to the up-regulation of phagocytosis, antigen processing and presentation in DCs and macrophages driving production of the Th1 phenotype of CD4+ T cells [19]. The relative importance of each of this diverse array of downstream effects on EBOV infection still needs to be elucidated.

EBOV evades the host innate immune signaling pathways through impaired type I and type II IFN signaling, dysregulated proinflammatory cytokine expression and suppression of DC maturation and T-cell activation [65–67]. This immune dysregulation is thought to allow EBOV to gain the upper hand during the course of infection; however, a reduction in viral antigen by as little as 2-fold appears to be the difference between the host survival and death during EBOV infection [68, 69]. As our IFNγ treatment of EBOV infected mice as late as 24 hours following lethal challenge demonstrates effective control of viral load, IFNγ treatment likely assists in overcoming EBOV-induced impairment of immune function by activation and maturation of immune cells as well as eliciting ISGs, leading to reduced EBOV RNA production as well as perhaps innate immune control of other aspects of the EBOV life cycle.

Filovirus pathogenesis has been investigated through the use of a variety of different animal models, including mice, guinea pigs, hamsters, and non-human primates (NHPs) [70–72]. These various model organisms offer different advantages for studying EBOV pathogenesis and assessing novel antiviral treatments. While NHPs are considered the most representative model for EBOV infection as they display very similar symptoms to those observed in humans [72], they are expensive and, ethically, the use of these animals should be limited to late phase pre-clinical studies. In contrast, infection of mice with MA-EBOV serves as a good, genetically manipulatable small animal model for early phase evaluation of vaccines and therapeutic interventions, allowing assessment of their impact on viral pathogenesis. Our definitive evidence of IFNγ efficacy in this small animal model paves the way for future NHP studies to further assay the anti-EBOV properties of IFNγ.

These *in vivo* studies suggest that IFNγ may serve as an effective prophylactic and/or therapeutic drug against EBOV infection. Several different IFNs are currently FDA approved to treat a variety of infections and autoimmune disorders. Type I IFNs have been used clinically as therapeutics against both hepatitis B and C infections [73, 74], but showed mixed efficacy against EBOV infection in NHPs [11–13]. The antiviral effects of IFNγ are less well-studied, but this protein is a FDA-approved therapy for chronic granulomatous disease and osteopetrosis [75, 76]. Chronic granulomatous patients receive IFNγ three times weekly to prevent infections, which can serve as life-long protective treatment in these patients [77, 78]. The established safety and effectiveness of IFNγ against these disorders and the ability of IFNγ to profoundly inhibit EBOV infection in our studies suggest that IFNγ may serve as an EBOV antiviral therapy.

## Materials and Methods

### Ethics statement

The University of Iowa Institution Animal Care and Use Committee (IACUC) guidelines were followed for all animal experiments and breeding that took place at the University of Iowa. These guidelines are in strict adherence to the Public Health Service Policy on Humane Care and Use of Laboratory Animals and the University of Iowa OLAW Assurance Number is A3021-01. All animal studies performed at the University of Iowa in this study were approved by the University of Iowa IACUC under protocol #1203072.

Texas Biomedical Research Institute (TBRI) Institution Animal Care and Use Committee (IACUC) guidelines were following for all animal experiments that took place at TBRI. These guidelines are in strict adherence to the Public Health Service Policy on Humane Care and Use of Laboratory Animals and the TBRI OLAW Assurance Number is A3082-01. All animal studies performed at TBRI in this study were approved by the TBRI IACUC under protocol #1445MU1.

### Mice

BALB/c mice were obtained from the National Cancer Institute (Frederick, MD) and BALB/c IFN-α/β receptor-deficient (IFNAR^-/-^) mice were a kind gift from Dr. Joan Durbin, NYU Langone Medical Center. Wild-type C57BL/6 and IFNγ receptor-deficient (IFNγR^-/-^) mice were a kind gift from Dr. John Harty, University of Iowa. Mice were bred at the University of Iowa.

Infectious EBOV mouse studies were performed in a biosafety level 4 laboratory (BSL4) at TBRI. For these studies, female BALB/c mice were obtained from Jackson Laboratory (Bar Harbor, ME) and housed under specific pathogen-free conditions.

### Production of virus

Recombinant wild-type EBOV expressing GFP for *in vitro* macrophage studies was generated as previously described [79]. Mouse adapted EBOV (MA-EBOV) was generated for *in vivo* mouse infections as previously described [80].

The production of recombinant, replication-competent vesicular stomatitis virus expressing eGFP and EBOV GP in place of the G glycoprotein (EBOV GP/rVSV) was performed as previously described [23, 25, 81]. The EBOV GP used in these studies was a GP lacking the mucin domain of GP1, which confers the same tropism as the full-length EBOV GP and produces higher titers [82–84]. EBOV GP/rVSV stocks were produced by infecting Vero cells at a low multiplicity of infection (~0.001) and collecting supernatants 48 hours following infection. Virus-containing supernatants were filtered through a 0.45 μm filter and stored at -80°C. EBOV GP/rVSV for mouse studies was concentrated by centrifugation at 7,000 x g at 4°C overnight. The virus pellet was resuspended and centrifuged through a 20% sucrose cushion by ultracentrifugation at 26,000 rpm for 2 hours at 4°C. The pellet was resuspended in PBS, aliquoted, and frozen at -80°C until use.

The generation of infectious VSV expressing eGFP utilized *in vivo* studies was previous described [85, 86]. VSV stocks were produced in Vero cells, concentrated and purified as described above.

### Primary cell isolation and cytokine stimulations

For isolation of murine peritoneal cells, mice were sacrificed and cells were harvested by lavage using 10 mL of ice-cold RPMI 1640 medium containing 10% FBS. The recovered peritoneal cells were washed in cold media and plated in the presence of 50 ng/mL of murine M-CSF (BioLegend, 576402). Non-adherent cells were removed 48 hours after isolation by washing twice with PBS. These adherent cells were previously characterized to express the macrophage surface markers, CD11b and F4/80 [87]. At 24 hours prior to infection, media was replaced with fresh media containing 50 ng/mL of murine M-CSF or GM-CSF with and without 20 ng/mL murine IFNγ (Cell Sciences, CRI001B) and/or murine TNFα (BioLegend, 575202). Unless otherwise indicated in the figure legend, 20 ng/mL of IFNγ was used for all studies.

The University of Iowa Institutional Review Board (IRB) approved all procedures for blood draws after obtaining informed consent from all individuals and all studies involving humans were performed under the approved University of Iowa IRB protocol #200607708 to Dr. Monick. Peripheral blood mononuclear cells (PBMCs) were isolated from human blood using Ficoll-Hypaque (Sigma-Aldrich), and monocyte-derived macrophages (MDMs) were isolated by adherence as previously described [88]. Isolated MDMs were plated in Dulbecco’s modified Eagle medium (DMEM) (Gibco) media containing 10% fetal bovine serum (FBS) (Atlanta Biologicals), 10% Type AB human serum (Sigma), 1% penicillin/streptomycin (P/S) (Life Technologies) and 50 ng/mL of human M-CSF (R&D Systems). MDMs were differentiated for 6 days. After 6 days, media on the MDM cultures was replaced with media containing human M-CSF alone with or without the addition of 20 ng/mL of IFNγ and/or TNFα (R&D Systems), unless otherwise indicated in the figure legend.

Human alveolar macrophages were isolated by bronchoalveolar lavage from healthy, consenting human volunteers as previously described [89]. Following isolation, the Wright-Giemsa-stained cytocentrifugation protocol was utilized to differentiate and count the total number of alveolar macrophages in the preparation. All cell preparations had between 90 and 100% alveolar macrophages [26, 27]. Alveolar macrophages were cultured in RPMI 1640 media containing 10% human serum, 10% FBS, 1% P/S and 50 ng/mL human M-CSF (R&D Systems) and stimulated with 20 ng/mL of human IFNγ for 24 hours prior to infection.

### EBOV and EBOV GP/rVSV infections of macrophages

#### EBOV

BALB/c peritoneal macrophages were isolated by lavage as described above and plated in the presence of 50 ng/mL murine GM-CSF or M-CSF. For prophylactic treatment, cells were treated with 20 ng/mL of murine IFNγ and/or murine TNFα for 24 hours prior to infection. Cells were refreshed with media without the addition of cytokines and transferred to a BSL4 facility at TBRI at 72 hours after cell isolation and challenged with replication-competent EBOV encoding GFP [79] at an MOI of 0.05. Cells were analyzed 24 hours following infection as described previously [25]. Briefly, the cells were fixed and stained with Hoescht 33342 dye and imaged with a Nikon Ti-Eclipse microscope using the high-content-analysis software package (Nikon, Tokyo, Japan). Images were processed and nuclei identified by Hoescht dye fluorescence. Total cells and cells expressing GFP were then counted using Cell Profiler software (Broad Institute, MIT, Boston, Massachusetts) using customized pipelines that are available from R. Davey upon request. Data are expressed as percent GFP positive cells.

#### EBOV GP/rVSV

Cytokine-containing media was removed from the macrophages and replaced with RPMI 1460 and 10% FCS. For the end point dilution assays, human and murine macrophage populations were infected with 10-fold serial dilutions of EBOV GP/rVSV (at least 4 replicates per dilution). Infection was scored 24 hours following infection for GFP positivity using a fluorescent microscope. Virus titers were calculated as the 50% tissue culture infective dose (TCID50)/mL by the Reed-Meunch method [90].

For flow cytometric analysis, murine IFNAR^-/-^ peritoneal macrophage infections were infected at an MOI of 0.1 based on titering virus stocks on Vero cells. These infections were quantified by detecting GFP positivity using a FACS Calibur flow cytometer (BD Biosciences). Data were analyzed using FlowJo analysis software. Relative infection was quantified compared to M-CSF stimulated peritoneal macrophages that equaled 100%.

### Visualization of infected human alveolar macrophages

Alveolar macrophages were infected with EBOV GP/rVSV at an MOI of 5 at 48 hours following isolation and 18 hours after IFNγ stimulation. Cells were fixed with 4% paraformaldehyde at 24 hours following EBOV GP/rVSV infection and covered with coverslips using Vectashield mounting medium with DAPI (H-1200; Vector Laboratories, Burlingame, CA). Images of alveolar macrophages were acquired using a Leica truepoint-scanning spectral system (TCS SPE) confocal microscope with Leica Application Suite (LAS AF) interface (Leica Microsystems, Wetzler, Germany).

### Mouse cell RNA isolation and quantitative reverse transcriptase PCR

Murine macrophage cultures were isolated and maintained as described above. Cells were infected with EBOV GP/rVSV or MA-EBOV at an MOI of 0.1. When indicated, cells were incubated 15 minutes prior to and during infection with protein synthesis inhibitor cycloheximide (CHX) (Sigma) at a concentration of 10 μg/mL [91]. Total RNA was isolated using the mirVana miRNA isolation kit (Ambion-Life Technologies) at 2, 6, 14 or 24 hours following infection. RNA was quantified by Nanodrop (Thermo Scientific). Total RNA (300 ng) was reverse-transcribed to cDNA using random primers and M-MLV reverse transcriptase (Invitrogen) using manufacturer’s specifications. Quantitative reverse transcriptase polymerase chain reaction (qRT-PCR) was used to detect transcript levels. SYBR Green based quantitative PCR reactions (Applied Bioscience) were performed using specific primers to VSV (M or L), EBOV (NP or L) [92], proinflammatory cytokine/chemokines or ISGs and 2 uL of cDNA from each reaction. Primer sequences are available upon request. Expression levels were defined as the ratio between threshold cycle (Ct) values and the housekeeping gene, HPRT. The determined ratios were converted to log2 values. For the replication experiments, results are represented as fold change of log2 values calculated based on the 2 or 6 hours M-CSF value.

### Human macrophage RNA extraction and microarray analysis

RNA preparation, quality analysis and microarray analysis were performed as previously described [93]. Microarrays assessed genome-wide macrophage mRNA expression using the GeneChip Human Exon 1.0 ST Arrays (Affymetrix). Microarray data were assessed by paired t-test and limma statistical analysis using R statistical software (http://www.R-project.org). Data were assessed for quality using a normalized unscaled standard error (NUSE) analysis. The method of statistical analysis was a paired t-test for each array. Limma analysis was performed to compare both macrophage arrays [94]. A 2-fold change in gene expression and *p* value of 0.01 was considered significant. Gene pathway analysis was conducted using GeneGo/Metacore version 6.19 (Thompson Reuters).

### Gene validation and analysis

Six of the top genes identified in the MDM and alveolar macrophage microarray analysis were validated by qRT-PCR as described above and previously described [93]. Primers specific for each of the ISGs were used to assess mRNA levels. Expression levels were defined as a ratio between threshold cycle values and housekeeping gene, HPRT. Primer sequences are available upon request.

### Interferon-stimulated gene inhibition studies

ISG-RFP lentiviral constructs were a kind gift from Dr. Charles Rice (Rockerfeller University) and have been previously described [40]. HEK 293T cells stably expressing TIM-1 (H3 cells) [23], which are routinely tested for the absence of mycoplasma, were transfected with the ISG-RFP constructs at the indicated concentrations. Forty-eight hours following transfection, cells were infected with EBOV-GP/rVSV at MOI = 0.2. Infection was assessed using flow cytometry (BD FACSVerse) 24 hours following infection. Flow cytometry data were analyzed using FlowJo cytometry analysis software for percentage of RFP positive cells that were also GFP positive. Relative infection was determined based on infection of cells expressing RFP-control construct alone.

ISG inhibition of infectious EBOV virus was performed using Neon (Invitrogen) electroporated HeLa cells (Ambion, Austin, TX). Forty-eight hours following transfection, cells were infected with EBOV-GFP at MOI of 0.5. After 24 hours, infection was quantified as described previously [25]. Briefly, images were analyzed using Cell Profiler to quantify RFP and GFP positive cells. Data were analyzed with FCS Express analysis software and cells were gated based on the no infection control.

### IRF1 siRNA knockdown of EBOV GP/rVSV infection by exosome delivery

#### Isolation of exosomes

The day before exosome collection the culture medium of HEK 293T cells was replaced with DMEM supplemented with pre-spun FBS. To avoid contamination of FBS derived exosomes, FBS were spun at 100,000 x g for 2 hours. The culture supernatants from the HEK 293T cells were collected and the exosomes were isolated from the supernatant by differential centrifugation as described previously [95, 96]. In brief, the cell supernatants were cleared of intact cells and cell debris by centrifugation at 1000 x *g* for 10 minutes and 10000 x *g* for 30 minutes at 4°C, and then supernatant was subjected to filtration through a 0.22 μm Amicon filter. The supernatants were concentrated in a 100 kDa MWCO Centricon Plus-15 (Millipore) to 1–2 mL. The concentrated supernatants were spun at 100,000 x *g* for 70 minutes in TLA 100.3 rotor (Beckman Coulter) to pellet exosomes. The collected exosomes were washed twice in PBS. The recovery of exosomes was estimated by measuring the protein concentration using Bradford assay.

#### Exosomes labeling and confocal imaging

Exosomes were loaded with 1X CellMask Deep Red Plasma Membrane stain for 10 minutes at 37°C. Exosomes were then washed with PBS and centrifuged at 100,000 x *g* for 70 minutes in TLA 100.3 rotor (Beckman Coulter) to pellet exosomes and resuspended in culture medium.

For imaging, peritoneal macrophages (300,000 cells) were allowed to attach in eight-well chamber slides. Ten micrograms of exosomes loaded with CellMask were applied to peritoneal macrophages and imaged 24 hour following treatment. Exosome uptake was visualized by on a Leica TCS SP3 confocal microscope (Leica Microsystems Inc., Buffalo Grove IL). Cells were fixed in 2% paraformaldehyde and mounted with Vectashield with DAPI (Vector Laboratories Inc., Burlingame, CA) before imaging.

#### Loading of DsiRNA into exosomes

Dicer-substrate RNAs (DsiRNA) (IDT), 27mer duplex RNAs that have increased potency in RNA interference compared to tradition siRNAs [97], were loaded into exosomes by electroporation as described [98]. Briefly, exosome pellets were resuspended in electroporation buffer for siRNA loading. The electroporation mixture was prepared by mixing exosomes and DsiRNA in 1:1 (wt/wt) in electroporation buffer. The final concentration of exosomes in the mixture was 250 ng/μL. The mixture was electroporated in a 400 μL volume using 0.4-mm cuvettes at 400 mV and 125 μF capacitance with pulse time of 10–15 ms. Exosomes were aliquoted and frozen at -20°C until use.

#### Exosome IRF1 knockdown and EBOV GP/rVSV infections

BALB/c IFNAR^-/-^ peritoneal macrophages were isolated as described above and cultured 250,000 cells in a 96-well format in the presence of 50ng/mL M-CSF for 48 hours prior to addition of DsiRNA loaded exosomes. IRF1 or scrambled (Scr) siRNA loaded exosomes (2.5 μg) were added to macrophages in fresh media with fresh M-CSF with or without 20ng/mL IFNγ. Macrophages and exosomes were spinoculated by centrifugation at 700 x *g* at 4°C for 1 hour and then incubated at 37°C for 24 hours. Twenty-four hours later, all media, exosomes and cytokines were removed and macrophages were infected with fresh media containing EBOV GP/rVSV at an MOI of 0.1. Twenty-four hours following infection, cells were lysed as described above for RNA extraction. RNA isolation, cDNA synthesis and quantification of IRF1/VSV-L expression by qPCR were performed as described above. Expression levels were defined as the ratio between threshold cycle (Ct) values for IRF1 or VSV-L and the housekeeping gene, mouse HPRT, and is displayed as the log2 value of this ratio.

### Mouse infections

#### EBOV GP/rVSV and VSV infections

Five -to eight-week-old male BALB/c IFNAR^-/-^ mice were administered recombinant murine (rm)-IFNγ (1, 3.3 or 10 μg/mouse as noted in figure legends of specific studies) (Cell Sciences, CRI001B) or PBS by intraperitoneal (i.p.) or intramuscular (i.m.) injection. Twenty-four hours after IFNγ injection, mice were infected with 10^3^ infectious units of EBOV GP/rVSV or 10^2^ infectious units of VSV virus by i.p. injection. In parallel experiments, other mice were administered IFNγ 2, 6, 12 or 48 hours following EBOV GP/rVSV infection by i.p. injection. In the VSV studies, post challenge administration of IFNγ was given only at 2 hours following infection. Mice were weighed and scored for sickness daily. Clinical sickness scoring was as follows: 0, no apparent illness; 1, slightly ruffled fur; 2, ruffled fur, active; 3, ruffled fur, inactive; 4, ruffled fur, inactive, hunched posture; 5, moribund or dead. Results represent scores from at least 10 mice per group. All mouse infection studies were concluded at 10 or 12 days following infection due to surviving mice regaining any lost weight and having no signs of clinical illness.

#### MA-EBOV infections

Five-week-old female BALB/c mice were administered rm-IFNγ (Cell Sciences, CRI001B) at the indicated concentration or PBS by i.p. injection 24 hours prior to, at the time, 6 or 24 hours following virus challenge. All of the mice were injected i.p. with 1000 plaque forming units (PFU) of mouse-adapted EBOV [99]. Mice were observed daily for weight loss and clinical scores. All surviving animals were sacrificed at day 12 or 14.

### Serum/organ viral titers

#### EBOV GP/rVSV infections

Organs were harvested 2 days following infection from at least 4 mice from each treatment group. Mice were anesthetized with isofluorene to perform retro-orbital bleeds for serum. Mice were euthanized and perfused with 10 mL of PBS through the heart prior to harvesting organs. The total peritoneal cell population was isolated and lysed for qRT-PCR to detect viral RNA as described above. Organs were homogenized in PBS and titers were determined by end-point dilution as described above on mycoplasma-free Vero cells.

#### MA-EBOV infections

Four days following infection, three animals per group were sacrificed, sera was isolated from blood samples and stored at -80°C until use. Virus loads were quantified by culturing 10-fold serial dilutions of serum in PBS on mycoplasma free Vero-E6 cells seeded in 6 well plates. After a one hour incubation, the supernatant was replaced with DMEM containing methylcellulose with 2% FBS and cells were incubated at 37°C for 10 days. Cells were fixed in 10% formalin overnight and stained with crystal violet. Plaques were manually counted.

### Statistical analysis

No statistical method was used to predetermine sample size. Tissue culture experiments were performed at least in triplicate with at least three replicates per experiment. *In vivo* survival experiments were performed at least in duplicate with at least 7 mice per treatment group. MA-EBOV or EBOV GP/rVSV titer studies were performed with at least three mice for each treatment group. When possible for both *in vitro* and *in vivo* studies, the investigators or veterinary staff were blinded to group allocation during the experiment and when assessing the outcome. Mice or samples were randomly assigned to various treatment groups. All data points and animals were reported in results and statistical analyzes.

Statistical analyses were performed using GraphPad Prism software (GraphPad Software, Inc.). Results are shown as means ± standard error of the means (s.e.m.). Two-tailed, unpaired Student’s t-tests were used to compare experimental treatment group to no IFNγ control for the majority of the studies reported here. To assess variance in these studies, F tests to compare variance were performed in parallel to determine that variance was similar between groups. For protein translation inhibition studies, a one-way analysis of variance (ANOVA) was performed to compare the no IFNγ control to the different treatments followed by a Tukey post hoc multiple comparison analysis to determine statistical significance. For nonparametric data, Mann-Whitney U-test was used. Log-rank (Mantel-Cox) tests were used to analyze differences in survival. *P* values less than 0.05 were considered significant.

## Supporting Information

S1 Fig IFNγ increases expression of proinflammatory cytokines IL-6 and TNFα and chemokines, CXCL10. M-CSF treated BALB/c peritoneal macrophages were untreated or stimulated with IFNγ 24 hours prior to infection. A subset of macrophages was infected with EBOV (MOI = 0.1) under BSL-4 conditions. Total RNA was harvested and cytokine/chemokine RNA expression quantified by qRT-PCR. Results represent means ± s.e.m. Data were analyzed by Student’s t-test compared to M-CSF control, *p < 0.05, **p < 0.01; ns, not significantly different.
(TIF)

S2 Fig IFNγ, not TNFα, inhibits EBOV GP/rVSV infection of BALB/c and C57BL/6 murine macrophages. (A) M-CSF-treated BALB/c peritoneal macrophages were stimulated with IFNγ, TNFα or the combination for 24 hours prior to EBOV GP/rVSV (MOI = 0.1) infection. Infection was quantified 24 hours following addition of virus by GFP positivity of the culture. (B) IFNγ, but not TNFα, inhibits EBOV GP/rVSV infection of M-CSF-treated C57BL/6 peritoneal macrophages. TCID_50_ values were determined by 10-fold serial dilutions of virus on to the macrophage cultures and titers assessed at 24 hours of infection by end-point dilution. Results represent means ± s.e.m. Data were analyzed by Student’s t-test compared to M-CSF-treated cells, **p < 0.01, ***p < 0.001, *p < 0.05.
(TIF)

S3 Fig IFNγ inhibition of EBOV GP/rVSV infection does not require the IFNα/β receptor, but does require the IFNγ receptor. (A) IFNγ inhibition of EBOV GP/rVSV infection is independent of the interferon α/β receptor (IFNAR) in BALB/c and C57BL/6 IFNAR^-/-^ peritoneal macrophages. M-CSF-treated cells were stimulated with IFNγ. Twenty-four hours later, cells were infected with EBOV GP/rVSV (MOI = 0.1) and assessed for GFP expression 24 hours following infection by flow cytometry. (B) Increasing concentrations of IFNγ block EBOV GP/rVSV (MOI = 0.1) in C57BL/6 IFNAR^-/-^ peritoneal macrophages in a dose-dependent manner. GFP expression was assessed at 24 hours by flow cytometry. (C) IFNγ receptor is required for IFNγ inhibition of EBOV GP/rVSV in mouse peritoneal macrophages. Peritoneal macrophages from C57BL/6 IFNγR^-/-^ mice were infected with 10-fold serial dilutions of EBOV GP/rVSV and virus titers were determined by end point dilution. Results represent means ± s.e.m. Data were analyzed by Student’s or one-sample t-test, *p < 0.05, **p < 0.01, ***p < 0.001. ns, not significantly different.
(TIF)

S4 Fig IFNγ inhibits EBOV GP/rVSV RNA production to the same extent as a protein synthesis inhibitor, CHX. Total RNA was isolated at 6 hours following EBOV GP/rVSV infection for qRT-PCR for VSV matrix (M) and polymerase (L) RNA. Results are represented as log2 values. Significance determined by ANOVA with a Tukey post-test. ***p < 0.01 (compared to M-CSF alone). ns, not significantly different.
(TIF)

S5 Fig Identification of IFNγ-responsive genes in human alveolar macrophages. (A) Differential gene expression profile of IFNγ-responsive genes in human alveolar macrophages. Genes were clustered into annotated ontology groups and listed in the corresponding GO groups table with their respective GO terms. Significance was determined by paired t-test analysis with cutoff values of at least two-fold change and *p* < 0.01. NUSE analysis of the array demonstrated that the means were centered at a value of 1 and minimum and maximum values between 0.95 and 1.05. ISGs that were assessed further in this study are bolded in the GO table. (B) mRNA validation of human alveolar macrophage profiling results for several of the most statistically significant IFNγ stimulated genes. RNA obtained for the microarray analysis was assessed for mRNA levels of the selected genes by qRT-PCR. Results are represented as the log2 values. Significance was determined by t-test analysis, *p < 0.05, **p < 0.01.
(TIF)

S6 Fig Identification of statistically significant genes identified in both gene arrays of IFNγ-stimulated human MDMs and alveolar macrophages. (A) A merged differential gene expression profile of IFNγ-responsive genes of our hMDM and human alveolar macrophage gene arrays. ISGs that were assessed further in this study are bolded. Significance was determined by limma analysis with cutoff values of at least two-fold change and *p* < 0.01. MDM, monocyte derived macrophages. AM, alveolar macrophages. (B) Identification of IFNγ differentially regulated gene pathways in macrophages. Representative bar graph displays the relative number of IFNγ responsive genes from both macrophage microarrays. Stripped bars, number of genes identified to correlate with each pathway that are represented in both the MDM and alveolar macrophage gene array datasets. Solid blue, genes unique to the MDM gene array results. Solid orange bars, genes unique to the alveolar macrophage gene array results.
(TIF)

S7 Fig HEK 293T exosomes are engulfed by murine peritoneal macrophages. Exosomes were isolated from HEK 293T cells and loaded with CellMask Deep Red Plasma Membrane stain. Exosomes (10μg) were washed and applied to BALB/c IFNAR^-/-^ peritoneal macrophages for 24 hours. Cells were washed, fixed and exosome uptake was visualized by confocal microscopy. DAPI stained nuclei (blue), actin (green) and exosomes (red).
(TIF)

S8 Fig IFNγ decreases morbidity and mortality associated with *in vivo* EBOV GP/rVSV and VSV infections. (A) IFNγ protects against weight loss by EBOV GP/rVSV infection at both 3.3 and 10 μg of murine IFNγ. Murine IFNγ (1, 3.3 or 10 μg) or PBS was administered 24 hours prior to infection with 10^3^ infectious units (iu) of EBOV GP/rVSV. Data represent 3 mice per group from 2 independent experiments. Death of mice at a particular day is indicated by a cross and value indicating the number of mice that succumb to infection. (B) IFNγ enhances survival of EBOV GP/rVSV infected mice and more modestly enhances survival following wild-type VSV infection. 3.3 μg IFNγ or PBS was administered by i.p. injection to BALB/c IFNAR^-/-^ mice 24 hours prior to or 2 hours following 10^3^ iu of EBOV GP/rVSV or 10^2^ iu VSV infection (n≥8/treatment). Protection studies with 3.3 μg of IFNγ were also performed at 6 hours following EBOV GP/rVSV challenge. Significance was determined by Mantel-Cox Test; compared to EBOV GP/rVSV PBS mice, ** *p*< 0.01, ***p < 0.001, compared to VSV PBS mice, # p< 0.05, ### p< 0.001, compared EBOV GP/rVSV IFNγ treated groups to VSV IFNγ treated groups, ∂ *p*< 0.05. (C) Treatment of mice with 10 μg IFNγ 24 hours prior to or 2, 6, 12 or 48 hours following EBOV GP/rVSV infection prevents weight loss during the first 3 days of EBOV GP/rVSV *in vivo* infection. (D) IFNγ (10 μg) treatment protects against weight loss at early days following infection. IFNγ was given 24 hours prior or 2, 6, 12 or 48 hours following EBOV GP/rVSV. For C and D, results represent means ± s.e.m. For B-D, treatment groups consist of at least 8 mice per group. Significance was determined by Student’s t-test compared to PBS control, ***p < 0.001.
(TIF)

S9 Fig IFNγ decreases morbidity and mortality associated with *in vivo* MA-EBOV infections. (A) Mice treated with 10 μg of IFNγ are protected from mortality associated with MA-EBOV infection. Doses of IFNγ or PBS were administered by i.p. injection to BALB/c mice 24 h prior to MA-EBOV infection. Significance was determined by Mantel-Cox Test, ***p < 0.001. (B) Weight loss of MA-EBOV infected mice following IFNγ treatment. Results represent means ± s.e.m. (C) Clinical sickness scores following MA-EBOV infection and IFNγ treatment. Findings in panels A-C represent 7 mice per group from one experiment. (D) IFNγ treatment inhibits MA-EBOV viremia. Serum was collected 4 days following infection and viral loads quantified with 10-fold serial dilutions of serum on Vero-E6 cells to determine PFU/mL. Data represent 2 or 3 mice per group. Significance was determined by Student’s t-test compared to PBS control, *p < 0.05.
(TIF)

S1 Table IFNγ stimulated genes significantly altered in human MDMs treated with 20 ng/ml of IFNγ as assessed by gene array analysis. ISGs that were assessed further in this study are bolded.
(XLSX)

S2 Table IFNγ stimulated genes significantly altered in human alveolar macrophages treated with 20 ng/ml of IFNγ as assessed by gene array analysis. ISGs that were assessed further in this study are bolded.
(XLSX)
