# Supplementary figures and images for: Interferon-γ Inhibits Ebola Virus Infection
Source: PLoS Pathog. 2015 Nov 12;11(11):e1005263. doi: 10.1371/journal.ppat.1005263 (PMC4643030; doi:10.1371/journal.ppat.1005263)

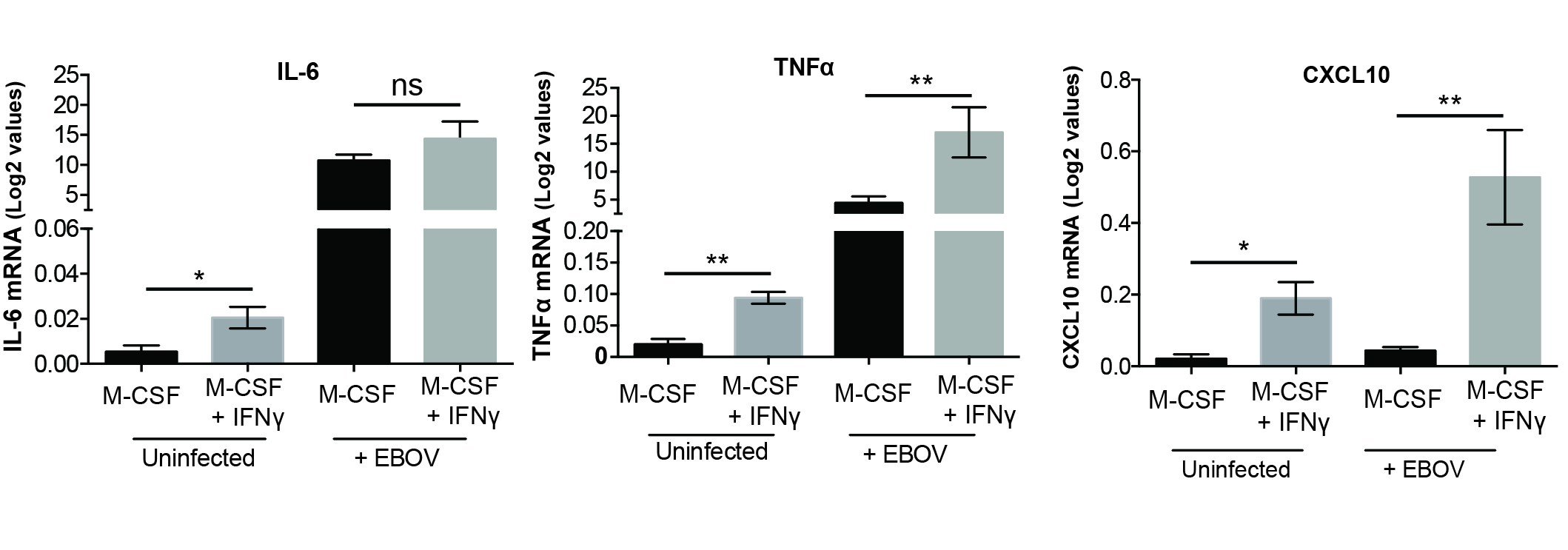

Supplement: S1 Fig — M-CSF treated BALB/c peritoneal macrophages were untreated or stimulated with IFNγ 24 hours prior to infection. A subset of macrophages was infected with EBOV (MOI = 0.1) under BSL-4 conditions. Total RNA was harvested and cytokine/chemokine RNA expression quantified by qRT-PCR. Results represent means ± s.e.m. Data were analyzed by Student’s t-test compared to M-CSF control, *p < 0.05, **p < 0.01; ns, not significantly different. (TIF) [file ppat.1005263.s001.tif]

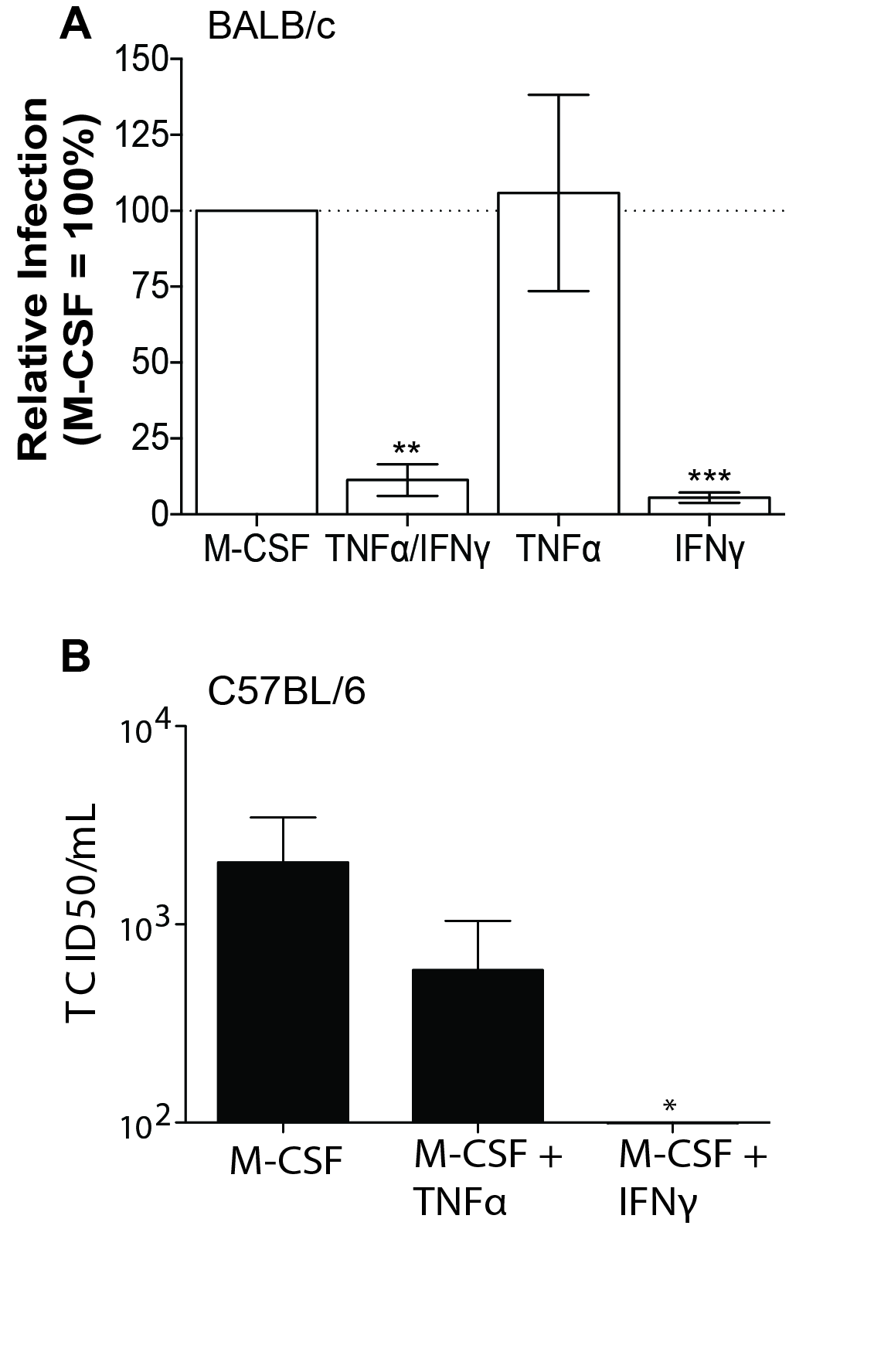

Supplement: S2 Fig — (A) M-CSF-treated BALB/c peritoneal macrophages were stimulated with IFNγ, TNFα or the combination for 24 hours prior to EBOV GP/rVSV (MOI = 0.1) infection. Infection was quantified 24 hours following addition of virus by GFP positivity of the culture. (B) IFNγ, but not TNFα, inhibits EBOV GP/rVSV infection of M-CSF-treated C57BL/6 peritoneal macrophages. TCID50 values were determined by 10-fold serial dilutions of virus on to the macrophage cultures and titers assessed at 24 hours of infection by end-point dilution. Results represent means ± s.e.m. Data were analyzed by Student’s t-test compared to M-CSF-treated cells, **p < 0.01, ***p < 0.001, *p < 0.05. (TIF) [file ppat.1005263.s002.tif]

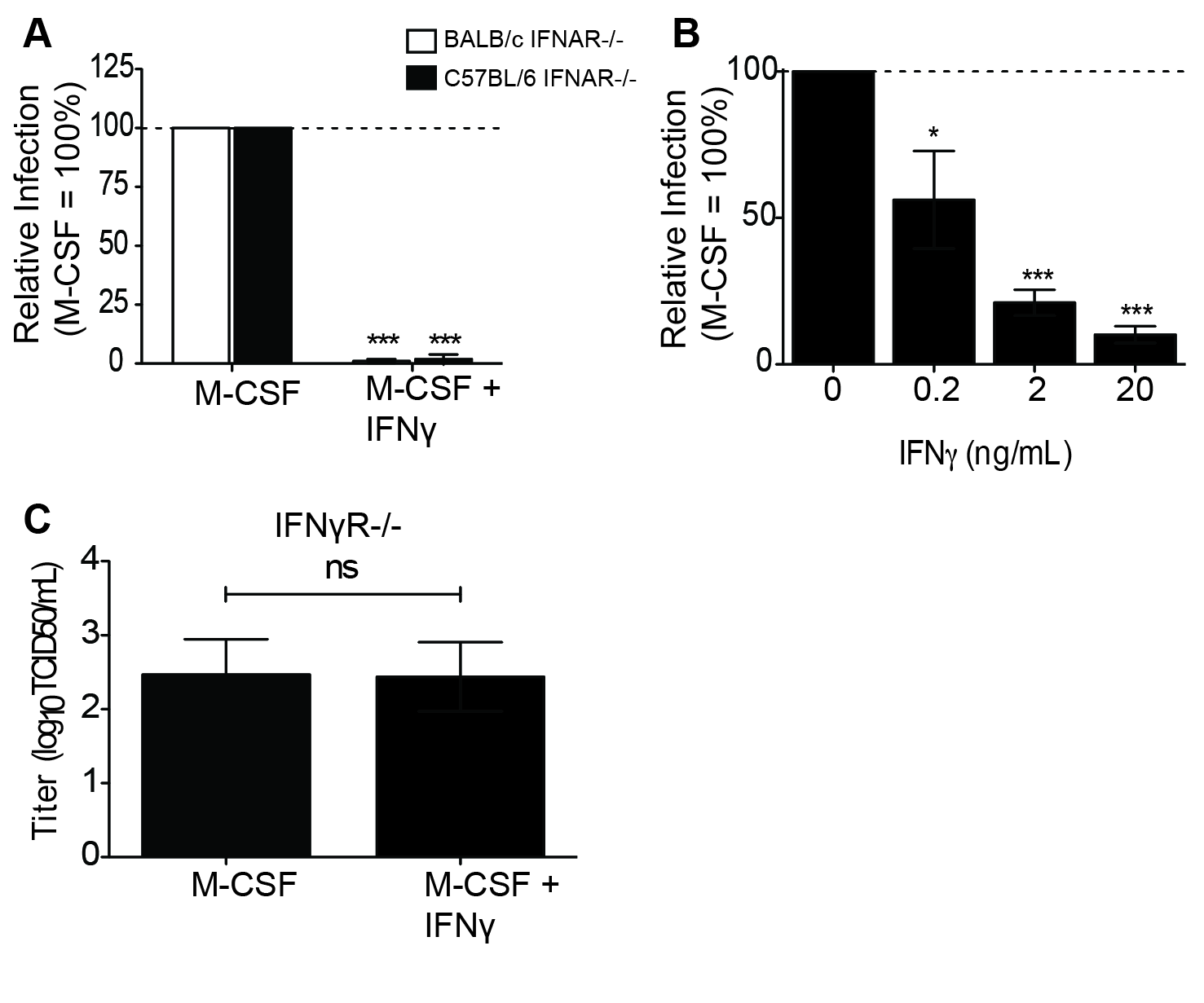

Supplement: S3 Fig — (A) IFNγ inhibition of EBOV GP/rVSV infection is independent of the interferon α/β receptor (IFNAR) in BALB/c and C57BL/6 IFNAR-/- peritoneal macrophages. M-CSF-treated cells were stimulated with IFNγ. Twenty-four hours later, cells were infected with EBOV GP/rVSV (MOI = 0.1) and assessed for GFP expression 24 hours following infection by flow cytometry. (B) Increasing concentrations of IFNγ block EBOV GP/rVSV (MOI = 0.1) in C57BL/6 IFNAR-/- peritoneal macrophages in a dose-dependent manner. GFP expression was assessed at 24 hours by flow cytometry. (C) IFNγ receptor is required for IFNγ inhibition of EBOV GP/rVSV in mouse peritoneal macrophages. Peritoneal macrophages from C57BL/6 IFNγR-/- mice were infected with 10-fold serial dilutions of EBOV GP/rVSV and virus titers were determined by end point dilution. Results represent means ± s.e.m. Data were analyzed by Student’s or one-sample t-test, *p < 0.05, **p < 0.01, ***p < 0.001. ns, not significantly different. (TIF) [file ppat.1005263.s003.tif]

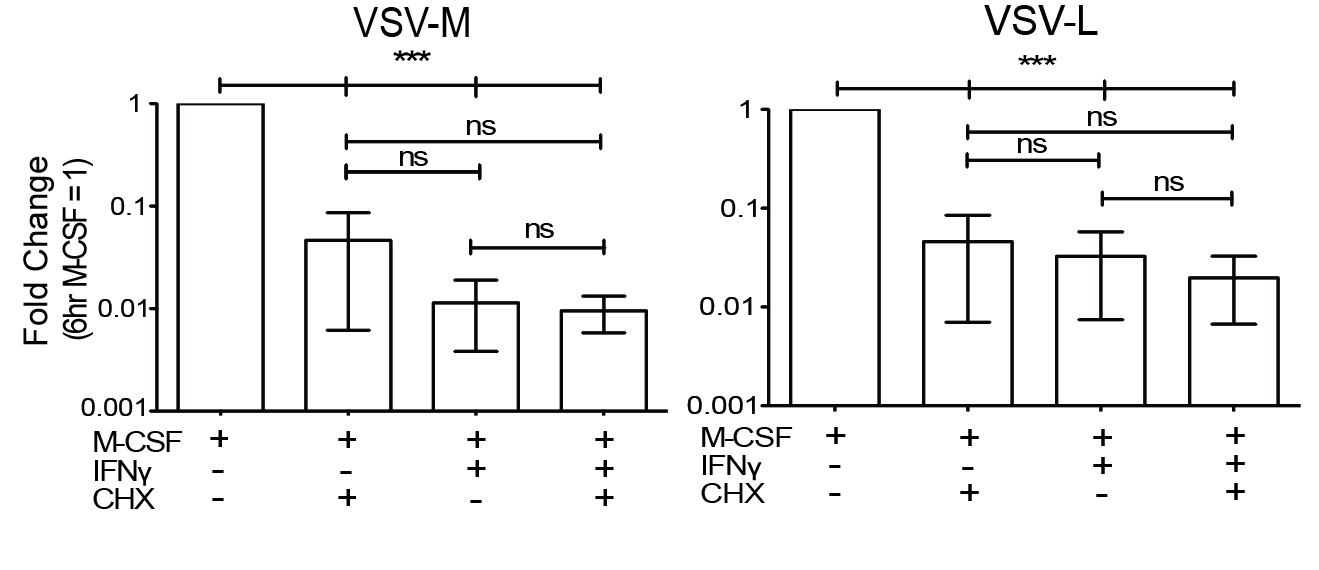

Supplement: S4 Fig — Total RNA was isolated at 6 hours following EBOV GP/rVSV infection for qRT-PCR for VSV matrix (M) and polymerase (L) RNA. Results are represented as log2 values. Significance determined by ANOVA with a Tukey post-test. ***p < 0.01 (compared to M-CSF alone). ns, not significantly different. (TIF) [file ppat.1005263.s004.tif]

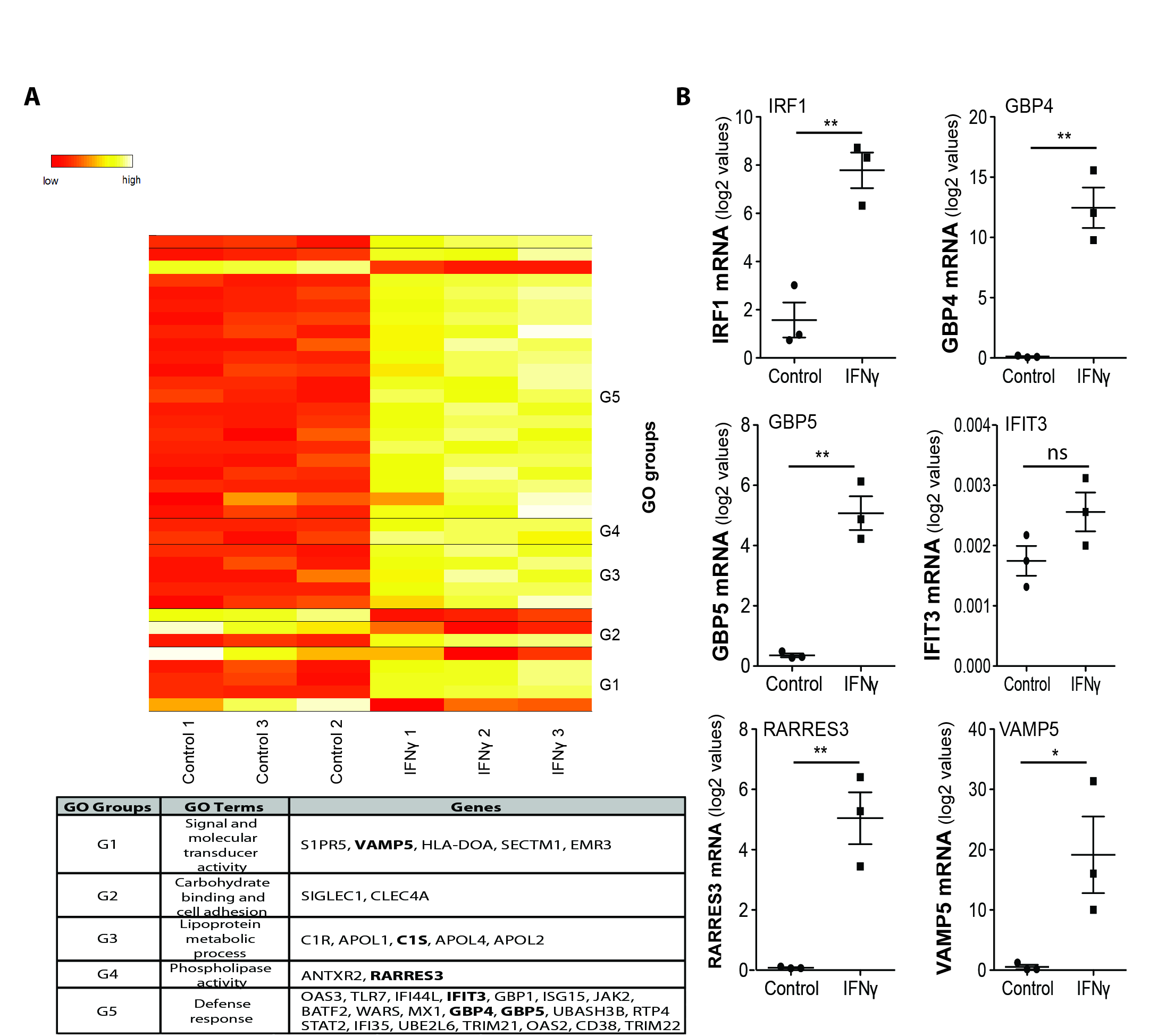

Supplement: S5 Fig — (A) Differential gene expression profile of IFNγ-responsive genes in human alveolar macrophages. Genes were clustered into annotated ontology groups and listed in the corresponding GO groups table with their respective GO terms. Significance was determined by paired t-test analysis with cutoff values of at least two-fold change and p < 0.01. NUSE analysis of the array demonstrated that the means were centered at a value of 1 and minimum and maximum values between 0.95 and 1.05. ISGs that were assessed further in this study are bolded in the GO table. (B) mRNA validation of human alveolar macrophage profiling results for several of the most statistically significant IFNγ stimulated genes. RNA obtained for the microarray analysis was assessed for mRNA levels of the selected genes by qRT-PCR. Results are represented as the log2 values. Significance was determined by t-test analysis, *p < 0.05, **p < 0.01. (TIF) [file ppat.1005263.s005.tif]

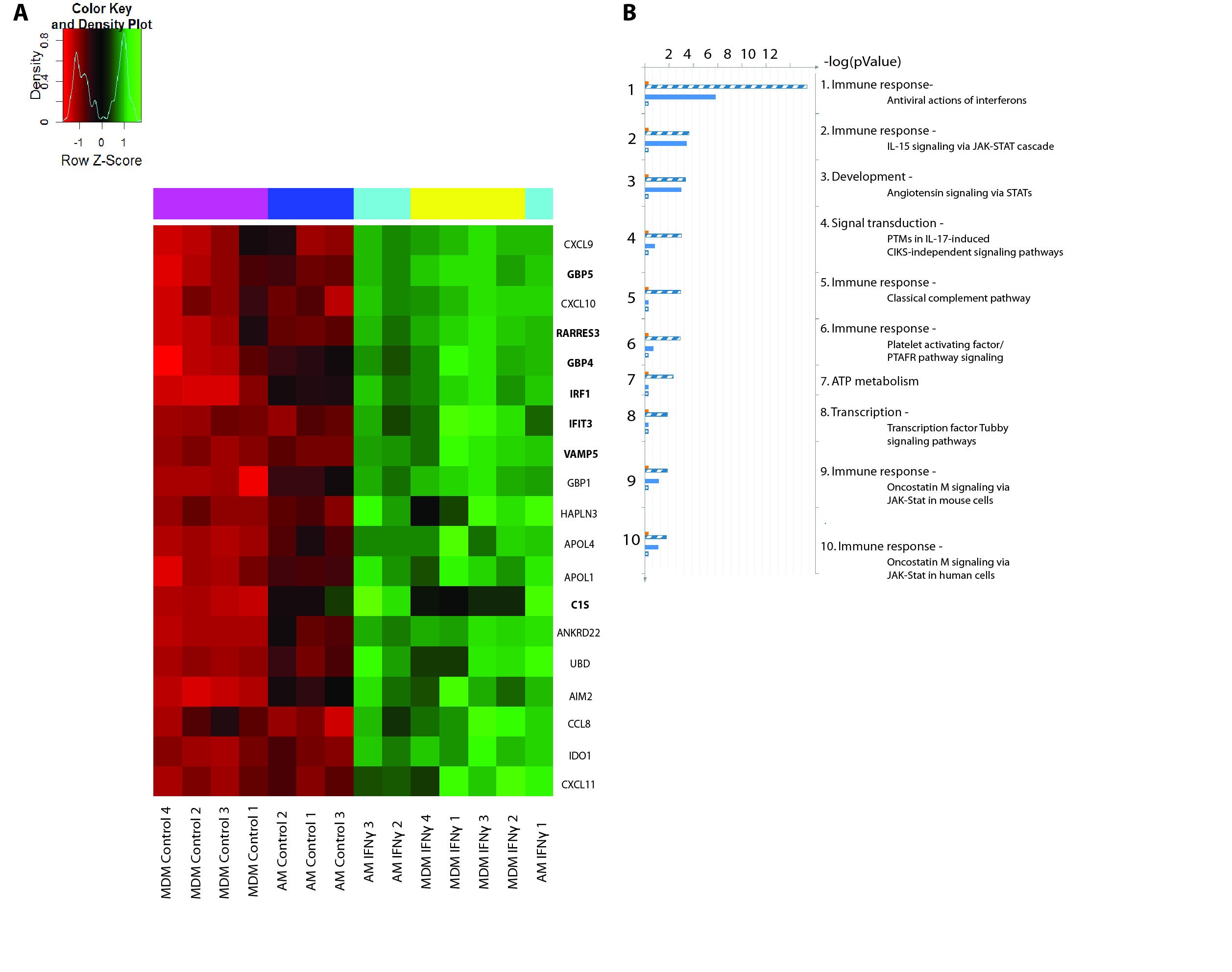

Supplement: S6 Fig — (A) A merged differential gene expression profile of IFNγ-responsive genes of our hMDM and human alveolar macrophage gene arrays. ISGs that were assessed further in this study are bolded. Significance was determined by limma analysis with cutoff values of at least two-fold change and p < 0.01. MDM, monocyte derived macrophages. AM, alveolar macrophages. (B) Identification of IFNγ differentially regulated gene pathways in macrophages. Representative bar graph displays the relative number of IFNγ responsive genes from both macrophage microarrays. Stripped bars, number of genes identified to correlate with each pathway that are represented in both the MDM and alveolar macrophage gene array datasets. Solid blue, genes unique to the MDM gene array results. Solid orange bars, genes unique to the alveolar macrophage gene array results. (TIF) [file ppat.1005263.s006.tif]

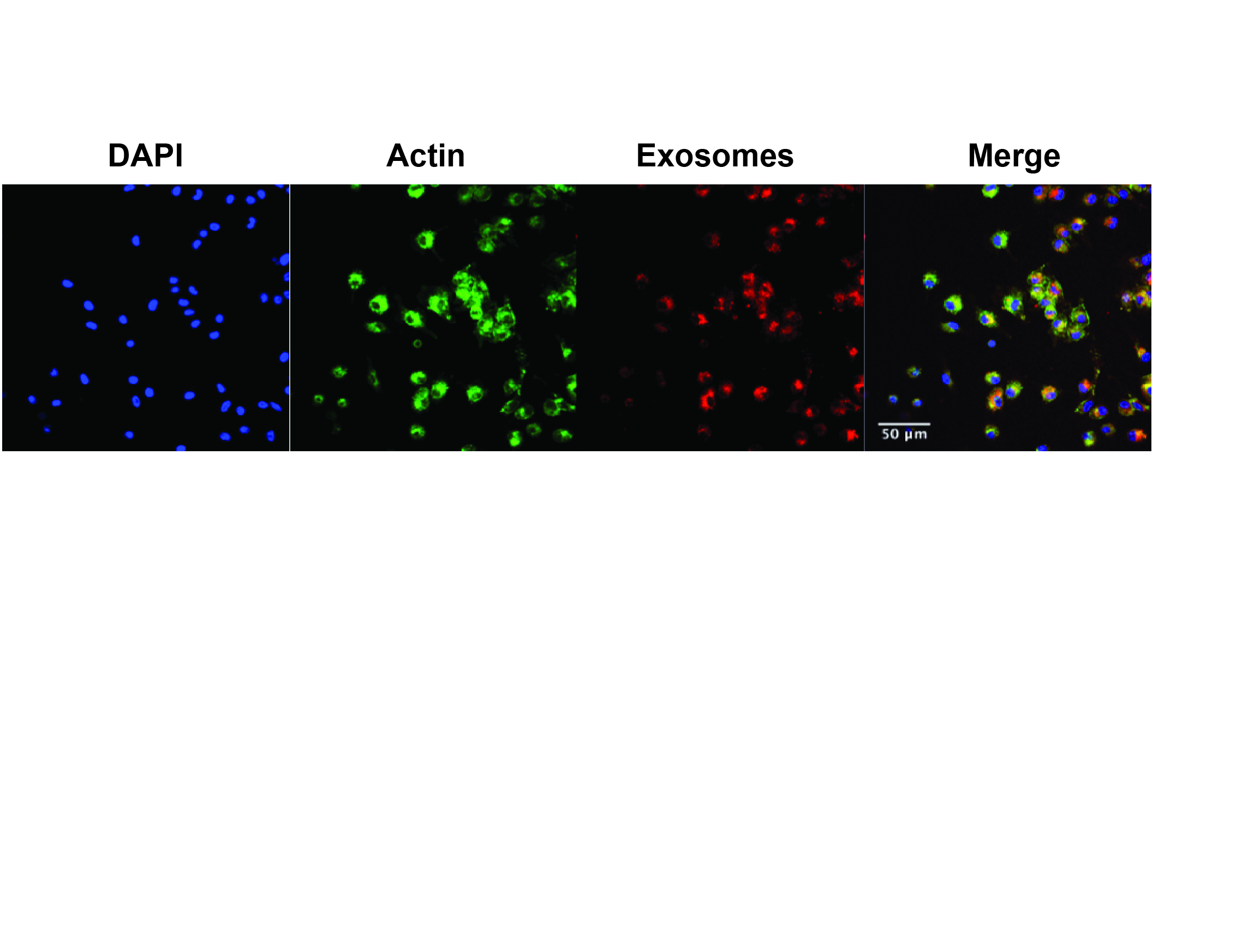

Supplement: S7 Fig — Exosomes were isolated from HEK 293T cells and loaded with CellMask Deep Red Plasma Membrane stain. Exosomes (10μg) were washed and applied to BALB/c IFNAR-/- peritoneal macrophages for 24 hours. Cells were washed, fixed and exosome uptake was visualized by confocal microscopy. DAPI stained nuclei (blue), actin (green) and exosomes (red). (TIF) [file ppat.1005263.s007.tif]

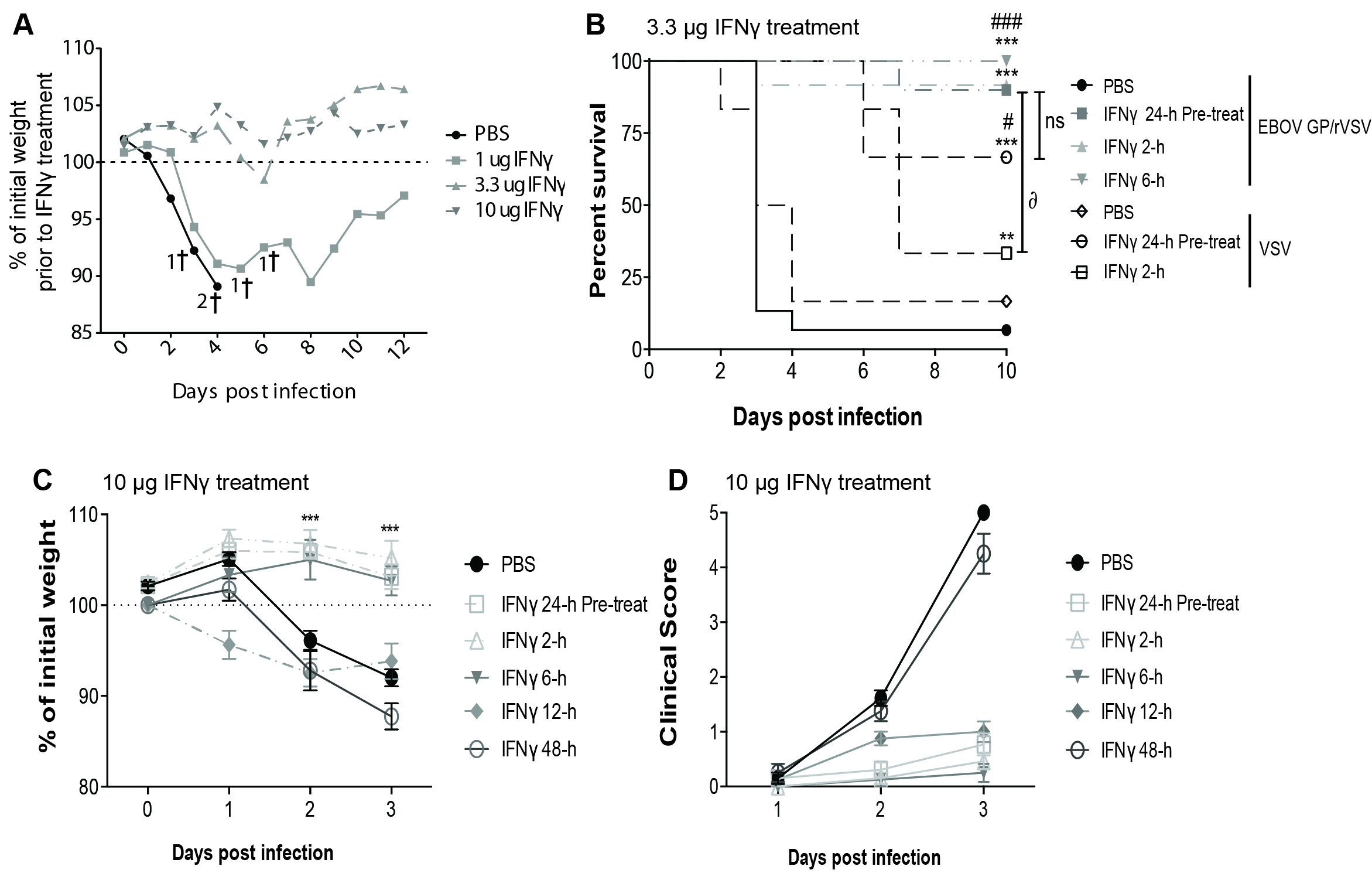

Supplement: S8 Fig — (A) IFNγ protects against weight loss by EBOV GP/rVSV infection at both 3.3 and 10 μg of murine IFNγ. Murine IFNγ (1, 3.3 or 10 μg) or PBS was administered 24 hours prior to infection with 103 infectious units (iu) of EBOV GP/rVSV. Data represent 3 mice per group from 2 independent experiments. Death of mice at a particular day is indicated by a cross and value indicating the number of mice that succumb to infection. (B) IFNγ enhances survival of EBOV GP/rVSV infected mice and more modestly enhances survival following wild-type VSV infection. 3.3 μg IFNγ or PBS was administered by i.p. injection to BALB/c IFNAR-/- mice 24 hours prior to or 2 hours following 103 iu of EBOV GP/rVSV or 102 iu VSV infection (n≥8/treatment). Protection studies with 3.3 μg of IFNγ were also performed at 6 hours following EBOV GP/rVSV challenge. Significance was determined by Mantel-Cox Test; compared to EBOV GP/rVSV PBS mice, ** p< 0.01, ***p < 0.001, compared to VSV PBS mice, # p< 0.05, ### p< 0.001, compared EBOV GP/rVSV IFNγ treated groups to VSV IFNγ treated groups, ∂ p< 0.05. (C) Treatment of mice with 10 μg IFNγ 24 hours prior to or 2, 6, 12 or 48 hours following EBOV GP/rVSV infection prevents weight loss during the first 3 days of EBOV GP/rVSV in vivo infection. (D) IFNγ (10 μg) treatment protects against weight loss at early days following infection. IFNγ was given 24 hours prior or 2, 6, 12 or 48 hours following EBOV GP/rVSV. For C and D, results represent means ± s.e.m. For B-D, treatment groups consist of at least 8 mice per group. Significance was determined by Student’s t-test compared to PBS control, ***p < 0.001. (TIF) [file ppat.1005263.s008.tif]

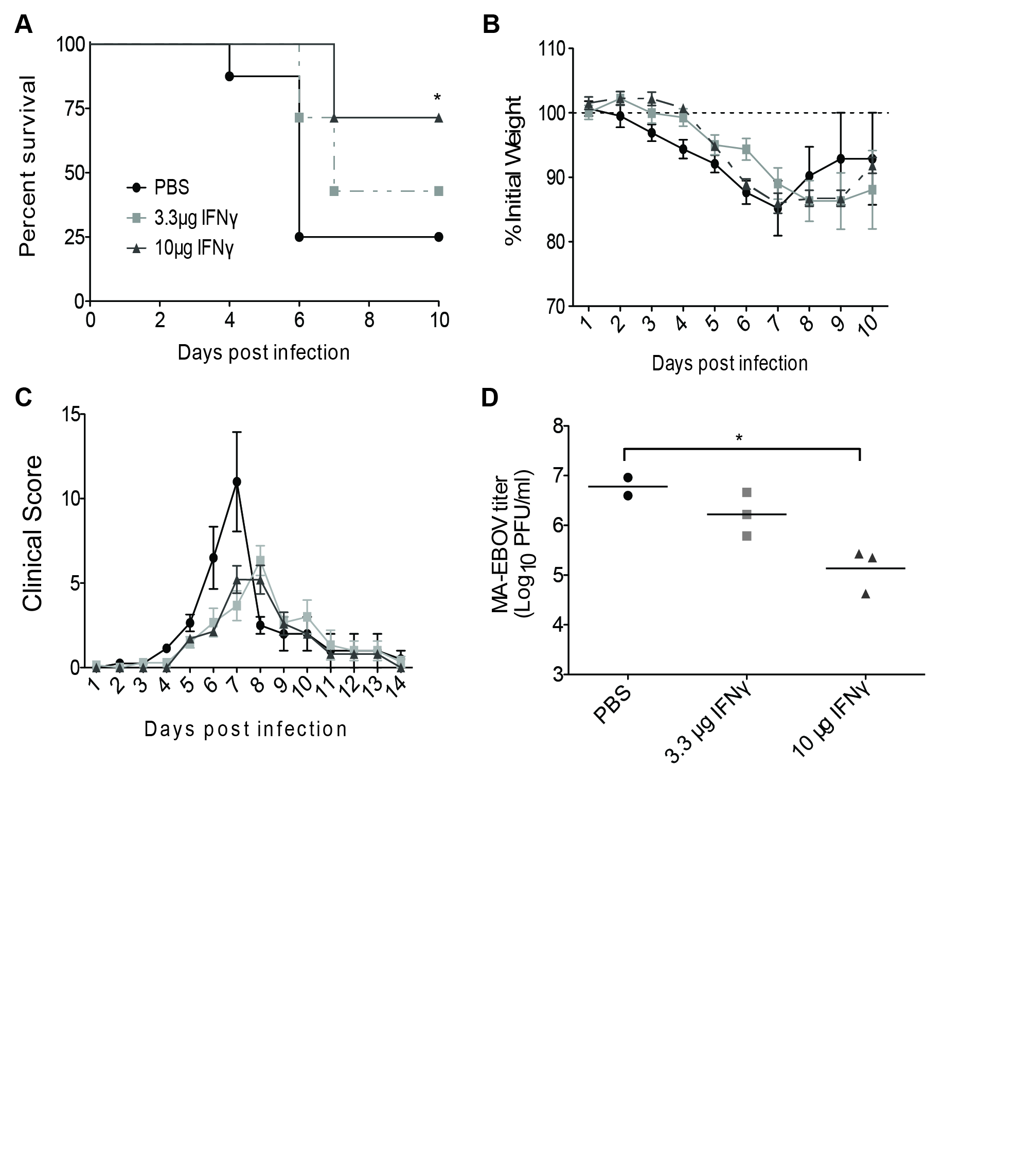

Supplement: S9 Fig — (A) Mice treated with 10 μg of IFNγ are protected from mortality associated with MA-EBOV infection. Doses of IFNγ or PBS were administered by i.p. injection to BALB/c mice 24 h prior to MA-EBOV infection. Significance was determined by Mantel-Cox Test, ***p < 0.001. (B) Weight loss of MA-EBOV infected mice following IFNγ treatment. Results represent means ± s.e.m. (C) Clinical sickness scores following MA-EBOV infection and IFNγ treatment. Findings in panels A-C represent 7 mice per group from one experiment. (D) IFNγ treatment inhibits MA-EBOV viremia. Serum was collected 4 days following infection and viral loads quantified with 10-fold serial dilutions of serum on Vero-E6 cells to determine PFU/mL. Data represent 2 or 3 mice per group. Significance was determined by Student’s t-test compared to PBS control, *p < 0.05. (TIF) [file ppat.1005263.s009.tif]
